# OLA1 regulates protein synthesis and integrated stress response by inhibiting eIF2 ternary complex formation

**DOI:** 10.1038/srep13241

**Published:** 2015-08-18

**Authors:** Huarong Chen, Renduo Song, Guohui Wang, Zonghui Ding, Chunying Yang, Jiawei Zhang, Zihua Zeng, Valentina Rubio, Luchang Wang, Nancy Zu, Amanda M. Weiskoff, Laurie J. Minze, Prince V.S. Jeyabal, Oula C. Mansour, Li Bai, William C. Merrick, Shu Zheng, Zheng-Zheng Shi

**Affiliations:** 1 Department of Translational Imaging Houston Methodist Research Institute, Weill Cornell Medical College, Houston, TX 77030, USA; 2 Cancer Institute, The Second Affiliated Hospital, School of Medicine, Zhejiang University, Hangzhou, Zhejiang 310009, China; 3 Department of Radiation Oncology Houston Methodist Research Institute, Weill Cornell Medical College, Houston, TX 77030, USA; 4 Department of Pathology and Genomic Medicine Houston Methodist Research Institute, Weill Cornell Medical College, Houston, TX 77030, USA; 5 Immunobiology Research Center Houston Methodist Research Institute, Weill Cornell Medical College, Houston, TX 77030, USA; 6 Biochemstry Department, Case Western Reserve University, Cleveland, OH 44106, USA

## Abstract

Translation is a fundamental cellular process, and its dysregulation can contribute to human diseases such as cancer. During translation initiation the eukaryotic initiation factor 2 (eIF2) forms a ternary complex (TC) with GTP and the initiator methionyl-tRNA (tRNAi), mediating ribosomal recruitment of tRNAi. Limiting TC availability is a central mechanism for triggering the integrated stress response (ISR), which suppresses global translation in response to various cellular stresses, but induces specific proteins such as ATF4. This study shows that OLA1, a member of the ancient Obg family of GTPases, is an eIF2-regulatory protein that inhibits protein synthesis and promotes ISR by binding eIF2, hydrolyzing GTP, and interfering with TC formation. OLA1 thus represents a novel mechanism of translational control affecting *de novo* TC formation, different from the traditional model in which phosphorylation of eIF2α blocks the regeneration of TC. Depletion of OLA1 caused a hypoactive ISR and greater survival in stressed cells. *In vivo*, OLA1-knockdown rendered cancer cells deficient in ISR and the downstream proapoptotic effector, CHOP, promoting tumor growth and metastasis. Our work suggests that OLA1 is a novel translational GTPase and plays a suppressive role in translation and cell survival, as well as cancer growth and progression.

Control of gene expression at the translational level allows cells to fine-tune protein synthesis and rapidly respond to extracellular stimuli, and plays a critical role in cellular homeostasis, differentiation, proliferation, and survival^1^,^2^,^3^. Under starvation or other stress conditions, cells use mechanisms of translational control, such as the integrated stress response (ISR), to conserve energy and reprogram gene expression^4^,^5^. During ISR, the α subunit of eIF2 is phosphorylated by four different eIF2α kinases in response to varied stresses: PKR (protein kinase R), PERK (PKR-like endoplasmic reticulum kinase), GCN2 (general control nonderepressible 2), and HRI (heme-regulated inhibitor). Phosphorylation of eIF2α (eIF2α-P) blocks the eIF2B-mediated exchange of eIF2-GDP to eIF2-GTP, limiting the regeneration of the eIF2 ternary complex (eIF2-GTP-Met-tRNA_i_^Met^, TC)^6^. With TC deficiency, global protein synthesis is suppressed, while activating transcription factor (ATF) 4 is preferentially translated, leading to cellular adaptation to the stress. During periods of prolonged stress, however, the ISR, along with sustained shutdown of essential protein synthesis and accumulation of ATF4-induced proapoptotic factors, can instead direct cells toward apoptosis^3^.

In fast growing carcinomas, cancer cells are constantly challenged by diverse stresses. Due to poor vascularization, tumors commonly outgrow their blood supply, leading to deprivation of oxygen, glucose and other nutrients^7^, and secondarily, to oxidative stress^8^ and endoplasmic reticulum (ER) stress^9^. As a result, high-level ISR is induced, as evidenced by the activation of eIF2α kinases and overexpression of ATF4^10^. Therefore, ISR mechanisms represent promising targets for anti-cancer therapy. To date, a number of different, even seemingly contradictory, strategies have been proposed to modulate tumor ISR. Interestingly, both ISR suppression^12^ and exaggeration^13^,^14^,^15^ have been reported to inhibit tumor growth *in vivo*. These paradoxical results may reflect the dual role of ISR in promoting either cell survival or apoptosis. Therefore, much remains to be learned about the regulation of ISR and its outcome.

In a search for additional ISR regulators among ancient GTPases, we pinpointed OLA1, a member of the Obg family of *P*-loop GTPases^16^,^17^,^18^,^19^, as a potent suppressor of mRNA translation and a key regulator of ISR. OLA1 was found to function via a novel mechanism that blocks the *de novo* formation of TC, in contrast to the well-characterized eIF2α-P-based mechanisms that limit the regeneration of eIF2-GTP. We further demonstrated that a hypoactive ISR status, mediated by knockdown (KD) of OLA1, was associated with the increased survival of cancer cells challenged with multiple stresses *in vitro*, and more strikingly, advanced tumor growth and metastasis *in vivo*. Finally, we observed a significant association between lower OLA1 expression and worse outcome in patients with breast cancer, indicating the significance of OLA1 in the prognosis of breast cancer.

## Results

### OLA1 is a negative regulator of mRNA translation in mammalian cells

**Figure 1:**
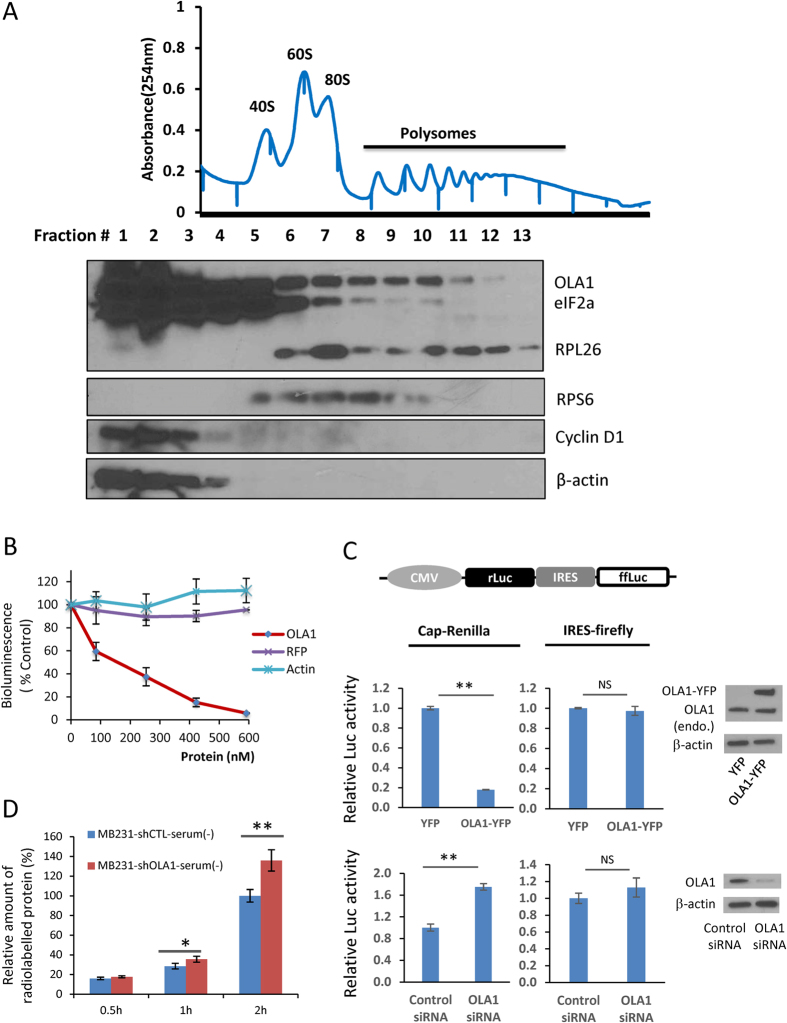
OLA1 suppresses mammalian protein synthesis. (**A**) Distribution of OLA1 in 10–50% sucrose density gradient fractions of A549 cell extract as measured by IB. Distributions of rpS6 (a small subunit protein), rpL26 (a large subunit protein), and eIF2α are shown for comparison. β-actin and Cyclin D1 were used as negative controls. Positions of the S40, S60, S80, and polysome fractions are also indicated. (**B**) The effect of OLA1 and control proteins (RFP and actin) on protein synthesis in RRL with luc mRNA as translation template (n = 3). (**C**) Effect of OLA1 overexpression and KD on mRNA translation *in vivo*. A diagram of the bicistronic reporter (rLuc-IRES-ffLuc which mediates the cap-dependent translation of Renilla luc and IRES-dependent translation of firefly luc) is shown (top panel). HeLa cells were transfected with OLA1-expression vectors (middle) or OLA1-siRNA (bottom) for 48 h, followed by transfection of the reporter for 24 h (n = 3). OLA1 expression was evaluated with IB (right). (**D**) Radiolabeling of *de novo* protein synthesis in OLA1-KD MDA-MB-231 cells. After 72 h starvation, serum was restored for indicated periods in the presence of [^35^S]Met/Cys (n = 3). Error bars: SD, Student’s t test: *p < 0.05; **p < 0.01; NS, not significant. Cropped blots are used. Full scan images of immunoblots are presented in Figure S10.

OLA1 belongs to the TRAFAC class, Obg family, and YchF subfamily of GTPases. Most of the TRAFAC GTPases are either associated with ribosomes or involved in translational control^17^. Polysome profiling of A549 and HEK293T cells revealed that OLA1 co-sedimented with ribosomal fractions (40S, 60S, 80S, and polysomes) (Fig. 1A and S1A). The role of human OLA1 in translation was tested using a rabbit reticulocyte lysate (RRL)-based *in vitro* system with luciferase (luc) mRNA as the translation template. Addition of wild-type (WT) OLA1 resulted in a significant dose-dependent decrease in luc activity (Fig. 1B). Neither of the control proteins (RFP or actin) had an effect on the bioluminescence. We further confirmed that the acquired luc activity indeed reflected the amount of the synthesized luc protein (Fig. S1B), and that OLA1 had no effect on luc activity after its synthesis (Fig. S1C). These data suggest that OLA1 acts as an inhibitory factor in protein synthesis.

Next we examined whether manipulation of OLA1 expression could modulate mRNA translation *in vivo* in HeLa cells. The rate of translation was assessed using a bicistronic luciferase reporter vector (rLuc-IRES-ffLuc), from which Renilla and firefly luciferases can be expressed through cap-dependent and HCV-derived internal ribosome entry site (IRES)-mediated initiation mechanisms, respectively. We found that ectopic expression of OLA-YFP caused an 80% decrease in Renilla luciferase activity but no significant change in firefly luciferase activity, as compared with the YFP vector control (Fig. 1C). On the other hand, OLA1-KD by transient siRNA transfection resulted in a 1.75 fold increase in Renilla luciferase, but not firefly luciferase (Fig. 1C). Together, these reporter assays underscored OLA1’s role in suppression of protein synthesis, specifically through a cap-dependent mechanism. To prove that OLA1 could affect synthesis of endogenous proteins, *de novo* protein synthesis was monitored by [^35^S] labeled methionine and cysteine (Met/Cys) incorporation. Indeed, OLA1-KD MDA-MB-231 cells released from serum starvation showed an overall increase in protein synthesis rate of ~25–35% as compared with the control cells (Fig. 1D). A similar enhancement of global protein synthesis during serum stimulation was also seen in HeLa cells with OLA1-KD (Fig. S1D). Both HeLa and MDA-MB-231 cell lines with deficient OLA1 exhibited increased amino acid (AA) restoration-stimulated global protein synthesis (Fig. S1E and S1F).

### OLA1 interacts with eIF2 and regulates its function

**Figure 2:**
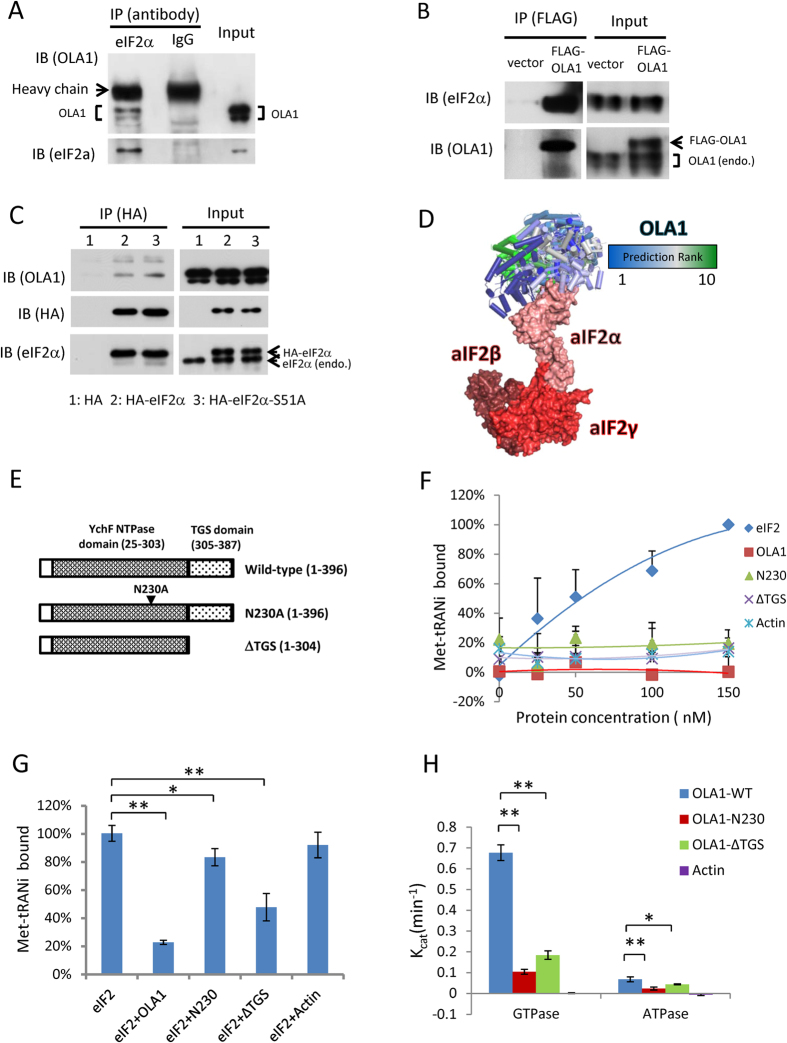
OLA1 binds to eIF2 and interferes with TC formation via its GTPase activity. (**A**) Interaction of endogenously expressed OLA1 and eIF2α in HEK293T cells. Cell lysate was immunoprecipitated with anti-eIF2α or anti-IgG antibody and the precipitates were immunoblotted with anti-OLA1 and anti-eIF2α antibodies. (**B**) Ectopically expressed OLA1 binds with endogenous eIF2α. HEK293T cells were transfected with a control or FLAG-OLA1 vector for 48 h. Cell lysates were subject to IP with anti-FLAG antibody and IB with the indicated antibodies. (**C**) Ectopically expressed eIF2α binds with endogenous OLA1. HEK293T cells were transfected with a control vector or expression vectors encoding HA-eIF2α or HA-eIF2α-S51A. Cell lysates were subjected to IP with anti-HA antibody and IB with indicated antibodies. (**D**) The top 10 predicted interactions between OLA1 (blue (#1)**→**green (#10); PDB ID: 2OHF)^16^ and aIF2 (red; PDB ID: 3CW2)^20^ as calculated by the program ZDOCK^48^. (**E**) Diagrams of the OLA1-WT and mutant proteins N230A and ΔTGS. (**F**) *In vitro* binding of eIF2 with [^14^C]-labeled yeast initiator Met-tRNAi^Met^ in the presence of GTP. The mean amount of Met-tRNAi bound to 150 nM eIF2 (~0.6 pmol) is set as 100%. (**G**) The effect of OLA1 proteins on formation of TC. Purified eIF2 was incubated with GTP and OLA1 (or other proteins), followed by addition of [^14^C]-Met-tRNAi^Met^. (Protein concentration: 150 nM for each protein.) (**H**) Nucleotide hydrolysis activity of the OLA1 proteins. Equal amount of protein (WT, N230A, ΔTGS, or actin; 1 μM) was incubated with [γ-^32^P]GTP or [γ-^32^P]ATP in reaction buffer at 35 °C for 30 min. The rate of the release of [^32^P]P_i_ was used to calculate K_cat_. Error bars: SD, Student’s t test: *p < 0.05; **p < 0.01. Cropped blots are used. Full scan images of immunoblots are presented in Figure S11.

Considering that OLA1 co-sediments with ribosomes/polysomes (Fig. 1A) and regulates cap-dependent mRNA translation (Fig. 1C), we explored the association of OLA1 with major initiation complexes^6^. Whereas the m^7^GTP-sepharose pull-down assay failed to identify an association of OLA1 with cap-binding complex (Fig. S2A), the interaction of OLA1 with the α subunit of eIF2 was established in HEK293T cells. OLA1 co-immunoprecipitated (IP) with both endogenously expressed eIF2α and ectopically expressed HA-tagged eIF2α, and reciprocally, ectopically expressed FLAG-tagged OLA1 co-IP-ed with endogenous eIF2α (Fig. 2A–C). The OLA1-eIF2α interaction was markedly increased in cells starved with AA for a short period (Fig. S2B). Direct binding of OLA1 with eIF2α was corroborated using an *in vitro* IP assay (Fig. S2C). To determine whether OLA1 binds the eIF2 holoprotein, another assay was performed using recombinant OLA1 as bait, which pulled down all 3 subunits (α, β, and γ) of eIF2 (Fig. S2D). While a complete eIF2 structure has yet to be solved, the structure of the archaeal homologue, aIF2, has been determined^20^. Consistent with a direct physical interaction, when the structure of OLA1 is computationally docked to aIF2, the most energetically favorable predicted interactions all occur with the aIF2α subunit (Fig. 2D).

To test whether OLA1 could interfere with the eIF2-mediated formation of TC, we measured the binding of purified eIF2 with [^14^C]Met-tRNAi^Met^ in the presence of GTP (Fig. 2E–G). Whereas co-incubation of OLA1-WT with eIF2 at equal molar concentrations severely suppressed the binding of Met-tRNAi^Met^ to eIF2 (80%), two mutant forms of the OLA1 protein, N230A (a point mutation at the G4 nucleotide binding motif)^16^ and ΔTGS (without C-terminal TGS domain), inhibited TC formation to a lesser extent (15% and 50%, respectively) (Fig. 2G). However, when the non-hydrolyzable GTP analog GDPNP was used instead of GTP, none of these proteins, including OLA1-WT, could prevent the binding of Met-tRNAi^Met^ to eIF2, suggesting GTP hydrolysis is necessary for OLA1’s mechanism of action (Fig. S3A). Consistently, when the WT and mutant OLA1 proteins were subjected to a [γ-^32^P]GTP-based GTPase assay, OLA1-WT showed substantial GTP hydrolyzing activity, whereas the N230A and ΔTGS mutations dramatically decreased this activity (Fig. 2H). Therefore, the inhibitory effect of OLA1 on TC formation appears to be attributable to its intrinsic GTPase activity.

However, OLA1 could not hydrolyze GTP bound to a previously formed TC, and co-incubation of OLA1 and eIF2 did not produce higher GTP hydrolysis than OLA1 alone, confirming that OLA1 and eIF2 were not acting as GTPase activating proteins toward each other (Fig. S3B and S3C). It is worth mentioning that in some earlier reports the YchF-sub family of GTPases was found to bind and hydrolyze ATP more effectively than GTP, and hence OLA1 was renamed as Obg-like ATPase^16^. However, our measurements indicate human OLA1 protein has a much stronger GTPase activity (K_cat_: 0.677 ± 0.038 min^−1^) than ATPase activity (K_cat_: 0.065 ± 0.012 min^−1^) (Fig. 2H).

Next, we asked if OLA1 could affect the phosphorylation of eIF2α. Addition of OLA1 protein to the RRL system had no effect on basal eIF2α-P, nor did it affect the phosphorylation of eIF2α by PERK (Fig. S3D). Additionally, an *in vitro* assay was employed to assess the effect of OLA1 on the PP1-mediated dephosphorylation of eIF2α, and it showed no effect (Fig. S3E).

### OLA1-KD attenuates ISR and renders cancer cells more resistant to multiple stresses

**Figure 3:**
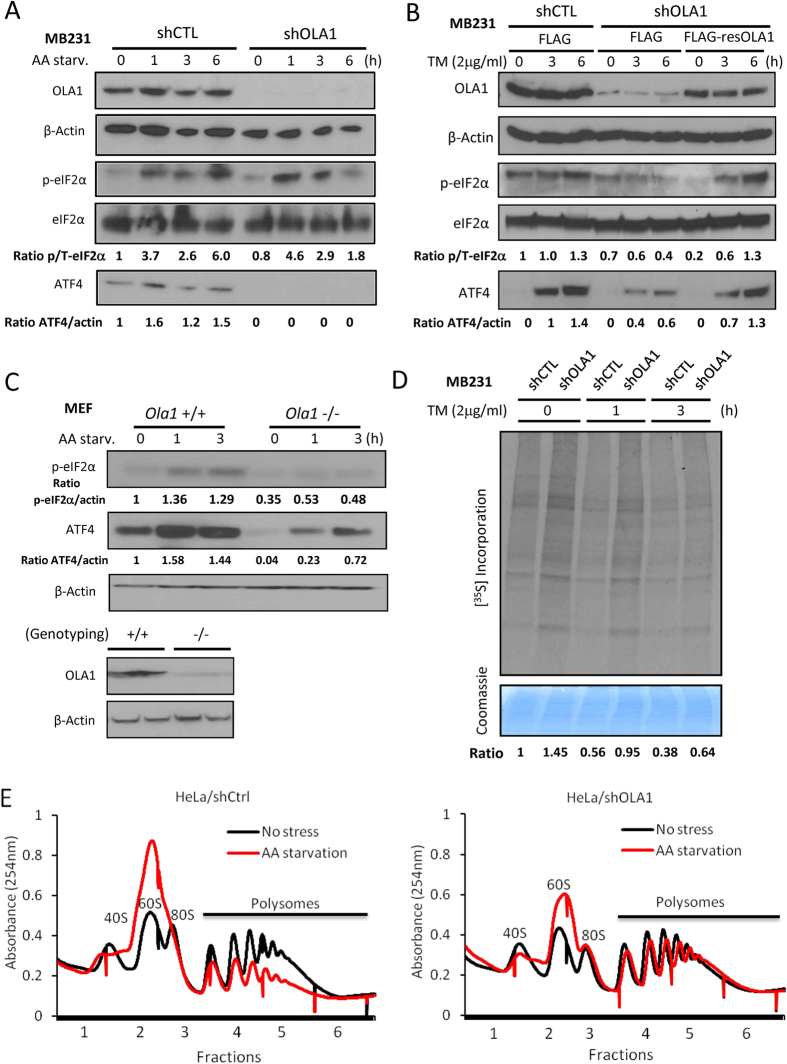
OLA1 regulates ISR signaling in cancer cells. (**A**) The shCTL and shOLA1 cells of MB231 origin were AA-starved for the indicated time and analyzed by WB. (**B**) Cells were transfected with FLAG-only or FLAG-resOLA1 plasmids for 48 h, then treated with 2 μg/ml TM. (**C**) *Ola1* (+/+) and (−/−) primary MEF cells were cultured in AA free medium for the indicated time. Genotyping of embryos used for MEF isolation was done by both PCR and IB (bottom). **(D)** Analysis of *de novo* protein synthesis in MDA-MB-231 shCTL and shOLA1 cells under ER stress. Cells were treated with TM for the indicated time and pulse-labeled with [^35^S]Met/Cys. Total proteins were separated on SDS-PAGE and autoradiographed. The [^35^S] incorporation (top) is normalized for loading of proteins indicated by Coomassie blue staining (bottom). **(E)** Polysome profiling of HeLa shCtrl and shOLA1 cells with or without 6 h AA starvation. Full scan images of immunoblots are presented in Figure S12.

Because eIF2 plays a major role in translational control in response to different stresses, we examined the induction of ISR in OLA1-KD cells. Paired cells stably transfected with OLA1 shRNA (shOLA1) and non-targeting shRNA (shCTL) were derived from HeLa and MDA-MB-231 (D3H2LN) cell lines. Total AA starvation was used to induce ISR and the accompanied activation of the eIF2α-P-ATF4 pathway (Fig. 3A). Compared to the shCTL cells, induction of ATF4 protein in the shOLA1 cells was markedly suppressed. When these shOLA1 and shCTL cells were exposed to other types of stresses, i.e., Tunicamycin (TM) for ER stress and H_2_O_2_ for oxidative stress, the characteristic impairment of ATF4 induction in OLA1-KD cells was reproduced (Fig. 3B and S4A). Under these conditions, eIF2α-P in the shOLA1 cells was induced to a lesser extent (Fig. S4A) or showed a faster recovery after induction (Fig. 3A). Moreover, when OLA1 expression was reconstituted in shOLA1 cells by transfection of shOLA1-resistant OLA1 cDNA (resOLA1), both the eIF2α-P and the induction of ATF4 were largely rescued (Fig. 3B and S4B). Additionally, when primary mouse embryonic fibroblasts (MEF)^21^ were tested with AA Starvation, the *Ola1* (−/−) MEFs showed deficiency in induction of eIF2α-P and ATF4 as compared with the *Ola1* (+/+) cells (Fig. 3C). To further evaluate the ISR status of these cells, we performed metabolic labeling of cells with [^35^S]Met/Cys. While the shCTL cells showed a progressive suppression of global protein synthesis after the TM treatment, the shOLA1 cells showed less suppression (Fig. 3D). Finally, we compared the polysome profiles of HeLa shCTL and shOLA1 cells in response to amino acid starvation. Notably, shOLA1 cells exhibited more resistance to the starvation-induced shift of polysomes to monosomes and ribosomal subunits (Fig. 3E). We thus concluded that the OLA1-KD cells had a hypoactive ISR in response to multiple stresses.

**Figure 4:**
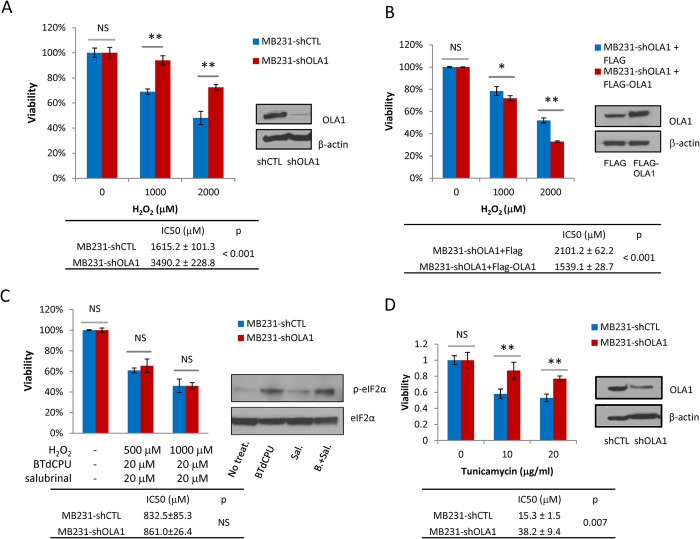
Cancer cells with downregulated OLA1 are more resistant to multiple stresses. (**A**) Cytotoxicity analysis of the MDA-MB-231 shCTL and shOLA1 cells exposed to different doses of H_2_O_2_ for 5 h. The OLA1 KD was verified by IB. (**B**) MDA-MB-231 shOLA1 cells were transfected with FLAG-only or FLAG-resOLA1 plasmid for 48 h then treated with H_2_O_2_ for 5 h. The re-expression of OLA1 was confirmed by IB. (**C**) MDA-MB-231 shCTL and shOLA1 cells were pretreated with 20 μM BTdCPU (N,N’-diarylurea) and 20 μM Salubrinal for 16 h then exposed to H_2_O_2_ for another 6 h. The status of eIF2α-P at the end of pretreatment was examined by IB. **(D)** Cytotoxicity analysis of the MDA-MB-231 shCTL and shOLA1cells exposed to different doses of TM for 5 h. Error bars: SD, Student’s t test: *p < 0.05; **p < 0.01; NS, not significant.

Consistent with our previous findings^22^, the shOLA1 cells showed increased resistance to oxidative stress compared to the shCTL cells, and this resistance could be reversed by restoration of OLA1 expression (Fig. 4A,B). These OLA1-KD cancer cells also showed increased survival under TM treatment, prolonged serum starvation, and total AA starvation (Fig. 4D, S5A and S5B). However, no notable changes in susceptibility were detected in these cells when challenged with hypoxia or glucose starvation (Fig. S5C–E). In order to verify that the downregulated ISR was an underlying mechanism for the pro-survival phenotype, we attempted to rescue the pathway using commercially available TC inhibitors: BTdCPU (N,N’-diarylurea), an activator of HRI that promotes eIF2α-P^14^, and Salubrinal, a selective inhibitor of eIF2α dephosphorylation^23^. We found that co-treatment with BTdCPU and Salubrinal could induce eIF2α-P substantially and stably. Pretreatment of the cells with BTdCPU and Salubrinal resulted in increased susceptibility of both the shOLA1 and shCTL cells to subsequent exposure to H_2_O_2_ (Fig. 4C). Under these conditions of TC-deficiency, OLA1-KD cells lose their survival advantage under oxidative stress.

### Downregulation of OLA1 has no or a negative impact on cell growth *in vitro* but promotes tumor growth *in vivo*

Under normal culture conditions, no discernible difference in cell growth was found between the stably transfected shOLA1 and shCTL cells of both HeLa and MDA-MB-231 origins (Fig. S6A and S6B). In contrast, Sun *et al.* reported that OLA1-KD human colon cancer cells (RKO and HT29) had a decreased rate of proliferation. This difference is probably due to tissue-type variations, because transient OLA1 knockdown in A549 (lung) cells did not affect cell growth (Fig. S6C), whereas in HK-2 (kidney) cells it led to a small decrease in proliferation (Fig. S6D). Using colony selection, several sub lines of MDA-MB-231 cells were developed (Approach 1), among which both of the shOLA1 clones (56E and 54F) showed a notably decreased growth rate compared to the two shCTL clones (C2B and C3F) (Fig. S6E). Thereby we concluded that depletion of OLA1 causes either no effect or a modest negative effect on cell proliferation.

**Figure 5:**
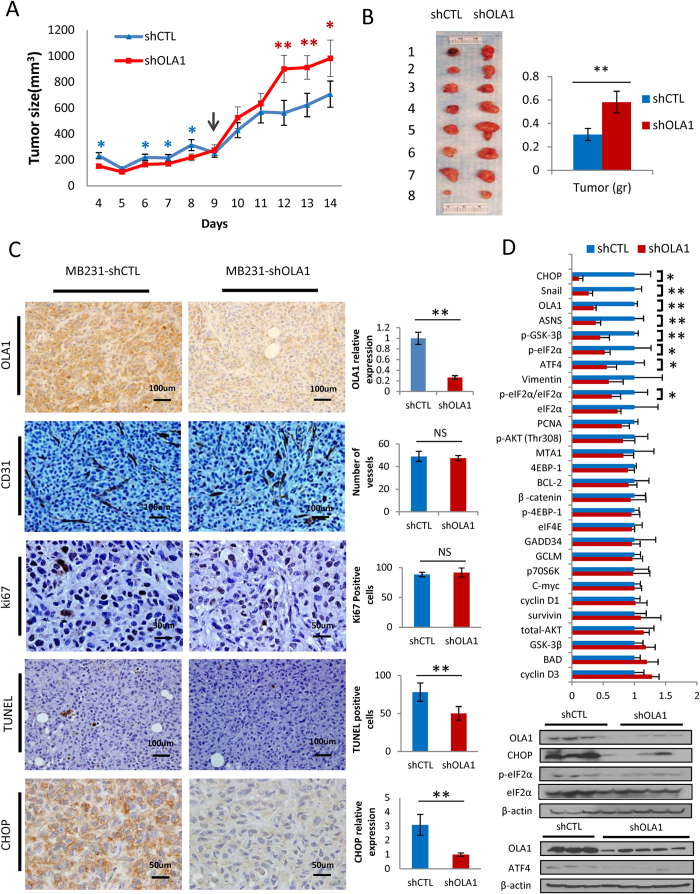
The effect of OLA1-KD on tumor growth in xenograft breast cancer models. (**A**) Growth curves of xenograft by inoculation of MDA-MB-231-derived (Approach 1) C2B (shCTL) cells into left and 56E (shOLA1) cells into right shoulder of the same nude mouse. Note that in early days the shOLA1 tumors were smaller (blue *), and after a “turning point” (arrow), bigger than the shCTL tumors (red *) (n = 8). (**B**) Photograph of the harvested tumors (left) and the weight of these tumors (right) (n = 8). (**C**) IHC analysis of tumor tissues harvested from an orthotopic breast cancer model (Approach 2, SCID mice). Representative microscopic images are shown for staining of OLA1 (cytosol), CD31 (vascular endothelium), Ki67 (nuclei), TUNEL (nuclei), and CHOP (cytosol). Quantitative analyses of each staining are shown as bar graphs on the right. At least 5 fields per slide and 3 slides per animal group were counted at 200× magnification. (**D**) IB analysis of tumor tissues harvested from the orthotopic breast cancer model (Approach 2, SCID). Levels of proteins and protein phosphorylation were quantified by ImageJ. The bar graphs (top) represent relative expression of each protein with mean values from the shCTL group set as 1.0 (n ≥ 3). IB (bottom) comparing the eIF2α-ATF4-CHOP pathway in shCTL and shOLA1 tumors. See also Figure S6 for all other IBs quantified in the bar graphs. Error bars: SEM, Student’s t test: *p < 0.05; **p < 0.01; NS, not significant. Cropped blots are used. Full scan images of immunoblots are presented in Figure S13.

We then performed several xenograft experiments to investigate the role of OLA1 in tumor growth *in vivo*. First, the cloned C2B and 56E cells were inoculated into left and right shoulders, respectively, of the same nu/nu mice. Interestingly, the OLA1-KD cells (56E) formed tumors that were smaller than the control cells (C2B) in the early days, but became significantly larger at a later phase (Fig. 5A). At the time of harvest, the average tumor weight of the 56E group was ~90% higher than the C2B group (*p* = 0.007) (Fig. 5B). Next, the MDA-MB-231 (D3H2LN)-derived shOLA1 and shCTL cells (without colony selection, Approach 2) were inoculated into 2 groups of SCID mice to induce orthotopic breast cancer. The shOLA1 group presented an elevated growth rate (*p* < 0.001), starting from mid-phase (Fig. S7A). Consistently, larger shOLA1 tumors were harvested (80% increase; *p* < 0.001). In all of these tumors the efficient OLA1-KD was shown by immunoblot (IB) analysis and immunohistochemistry (IHC) staining (Fig. 5C,D). Therefore, OLA1-KD tumors have superior growth potential in the xenograft environment regardless of the cells’ proliferative ability under culture conditions.

Using IHC staining, we examined the tumor tissues for an angiogenesis marker (CD31), proliferation marker (Ki67), and rate of apoptosis (TUNEL) (Fig. 5C). The number of CD31-positive vessels was comparable between the two tumor groups, ruling out angiogenesis as a contributing factor to the growth phenotype. There was also no difference in Ki67 expression. However, the number of apoptotic cells was significantly lower in the shOLA1 group than the shCTL group (*p* = 0.001), suggesting that the growth advantage was due to a net decrease in cell death without changes in proliferation. CHOP, a pro-apoptotic transcription factor and downstream effector of ISR, was found to be greatly diminished in the shOLA1 tumors (Fig. 5C,D).

The harvested tumor tissue lysates were subjected to IB analysis with over 30 antibodies (Fig. 5D and S7B). The most prominent molecular alterations in shOLA1 tumors occurred to the ISR pathway: a decreased ratio of P-Ser51/eIF2α and decreased ATF4, CHOP, and ASNS (asparagine synthase, an ATF4 target), together representing a hypoactive ISR status, strikingly consistent with our *in vitro* characterization of the OLA1-KD cells. Other significant alterations included the hypophosphorylation of GSK3β (Ser9) and the deficiency of one of its substrates, Snail, together indicating a hyperactive GSK3β. However, no notable changes were detected for Akt (PI3K/Akt signaling); c-myc (proto-oncogene); BCL2, Survivin, and BAD (apoptosis or anti-apoptosis associated); p70S6K, eIF4E, and 4EBP-1 (involved in protein synthesis); Cyclin D1, Cyclin D3, and PCNA (regulating cell cycle progression); or MTA1, β-catenin, and Vimentin (epithelial-mesenchymal transition related).

### Downregulation of OLA1 promotes metastasis in an animal model

**Figure 6:**
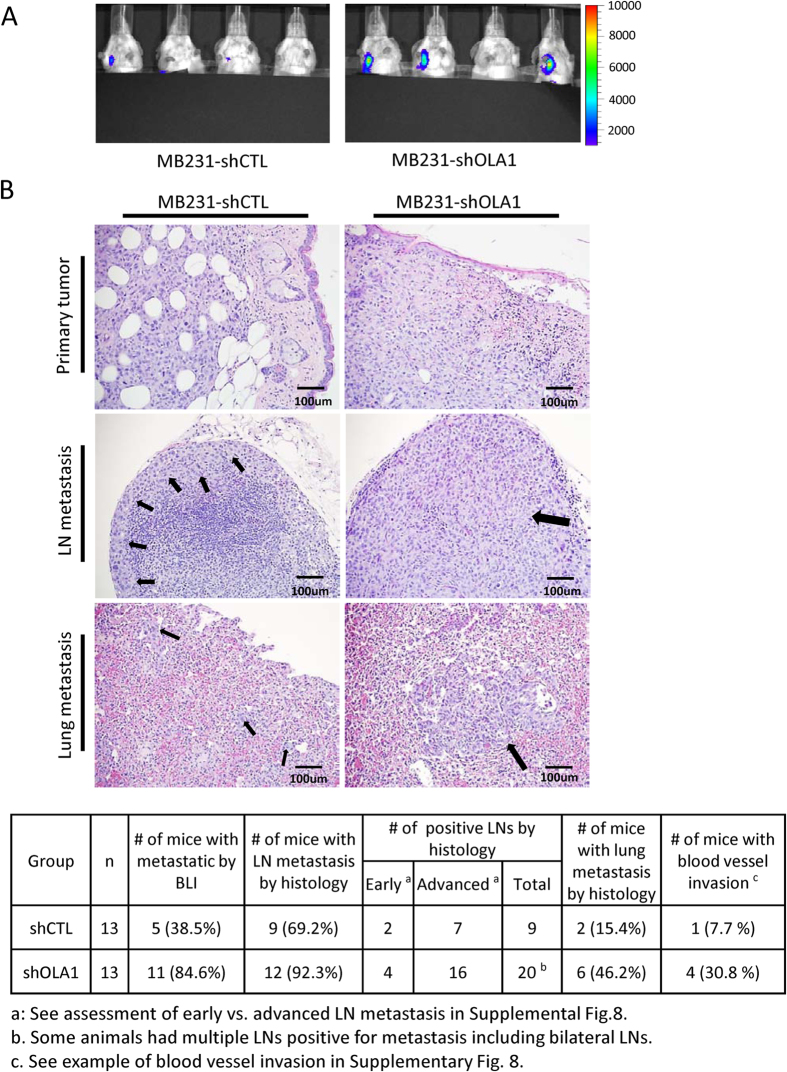
The effect of OLA1-KD on metastasis in orthotopic breast cancer models. (**A**) BLI of female SCID mice bearing MDA-MB-231 (D3H2LN) tumors in their mammary fat pads. Representative images of animals were taken on 30^th^ day after inoculation. Signals from the axillary/brachial regions indicate LN metastasis. The primary tumors were shielded due to their overwhelming brightness. (**B**) Histologic analysis of the primary tumors, LNs, and lungs dissected from the shCTL and shOLA1 groups. The arrows indicate the presence of tumor cells at LN and lung. Bottom: summaries of remote site metastasis as assessed by BLI and histopathology.

The MDA-MB-231 (D3H2LN) cell line is engineered with an imaging reporter (luc) and has a high potential to metastasize^24^, thus facilitating the monitoring of metastasis in orthotopic breast cancer models. Bioluminescent imaging (BLI)-positive lymph nodes (LNs) were detected in 11/13 animals from the shOLA1 group compared to 5/13 from the shCTL group, suggesting that OLA1-KD tumors developed more LN metastasis (Fig. 6). Subsequent necropsy and histopathology were conducted throughout the body. When additional criteria were applied, including early-stage vs. advanced metastasis (Fig. S8A), and total counts of positive LNs, the shOLA1 group was confirmed to carry more metastatic LNs by each category compared to the shCTL group. By serial sectioning of the lungs, 6/13 shOLA1 and 2/13 shCTL mice were confirmed with lung metastasis. In the primary tumors, we were able to identify disseminated cancer cells inside blood vessels in 4/13 shOLA1 and 1/13 shCTL mice, a phenomenon indicating the intravasation step of metastasis (Fig. S8B). Together, these imaging and histopathological data allowed us to conclude that tumors with OLA1-KD have an increased potential to metastasize.

### Lower expression of OLA1 correlates with worse prognosis in patients with breast cancer

**Figure 7:**
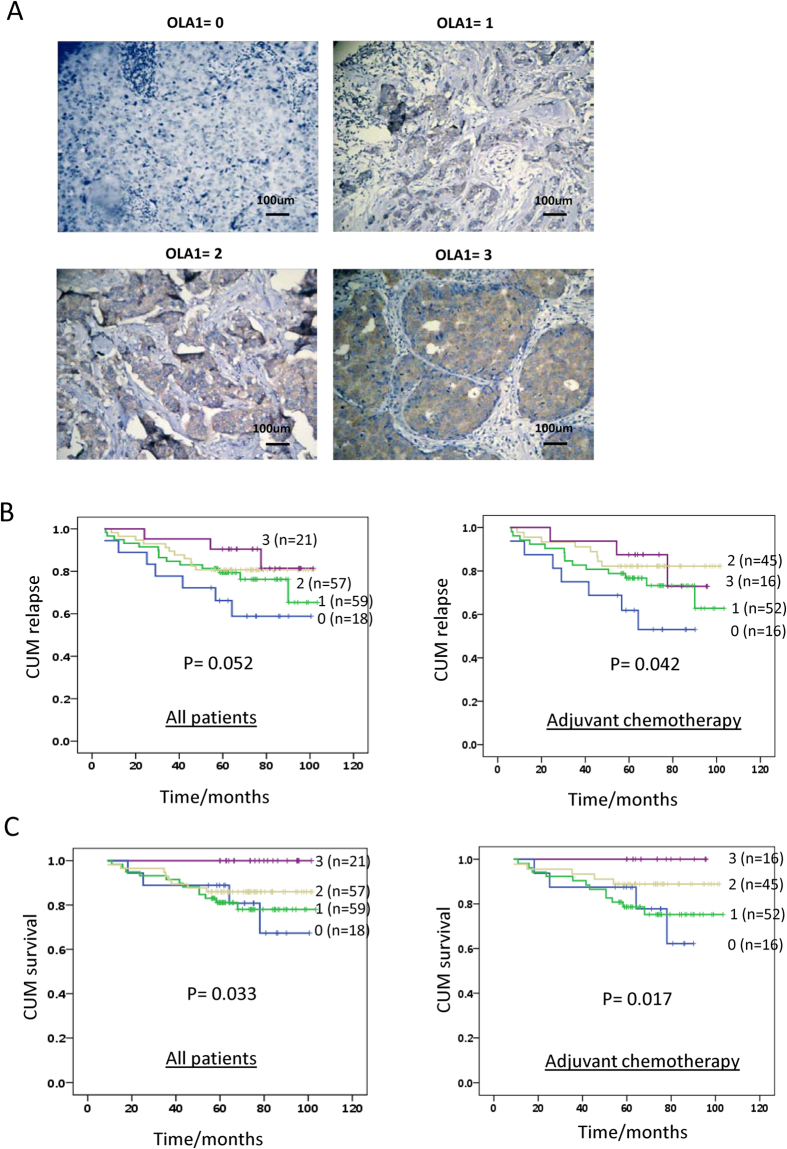
OLA1 is a potential prognostic biomarker for patients with breast cancer. (**A**) Representative micrographs of IHC staining of OLA1 in human breast cancer tissues. Expression of OLA1 was scored (0–3). (**B**) Kaplan-Meier analysis for breast cancer relapse in all patients (left panel) and those undergoing adjuvant chemotherapy (right panel). Lower expression of OLA1 indicates more relapse in patients. (**C**) Kaplan-Meier analysis for DSS in all patients (left panel) and those undergoing adjuvant chemotherapy (right panel). Lower expression of OLA1 indicates worse DSS in patients.

To address the correlation between OLA1 expression and clinical features of breast cancer, 160 patient cases were analyzed by IHC for OLA1. Patients were divided into 4 groups (0–3) based on the OLA1 levels (Fig. 7A). We compared OLA1 expression with clinical parameters including patients’ age, tumor size, histological type, pT (tumor invasion stage), and pN (LN metastasis) by ANOVA analysis, without finding a significant correlation (Supplementary Table 1). However, with Kaplan-Meier analysis, we found that lower OLA1 expression correlated with a higher chance of cancer relapse (recurrence or metastasis after surgery) and a decreased DSS (disease specific survival) (left panels of Fig. 7B,C, log-rank: *p* = 0.052 and 0.033, respectively), especially in those patients who received adjuvant chemotherapy (n = 129) (right panels of Fig. 7B,C, log-rank: *p* = 0.042 and 0.017, respectively). We further searched The Cancer Genome Atlas (TCGA, https://genome-cancer.ucsc.edu) for OLA1 expression pattern in breast cancer patients. As shown in Fig. S9A, both gene deletion (0.4%) and amplification (0.5%) was observed in 962 cases of breast cancer patients from TCGA cohort^25^,^26^. Interestingly, in the same cohort, lower OLA1 mRNA expression is associated with an increased depth of tumor invasion (*p* = 0.021) or metastasis (*p* = 0.051) in 1215 patients (Fig. S9). From these data, low OLA1 expression may represent a novel prognostic factor for breast cancer, especially for patients undergoing adjuvant chemotherapy.

## Discussion

The Obg family of GTPases is highly evolutionarily conserved from bacteria to humans^17^,^18^,^27^. Despite phylogenetic and structural analyses that hinted the YchF/OLA1 subfamily proteins are GTP-dependent translation factors^16^,^19^, the molecular basis for this putative function and its downstream effects has been poorly characterized except demonstrations of their association with ribosomes in *E. coli* and *T. cruzi*^28^,^29^,^30^. We present here experimental evidence that supports human OLA1, a cytosolic protein implicated in cellular stress responses^21^,^22^, as a key regulator of both protein synthesis and translational control in response to stress.

**Figure 8:**
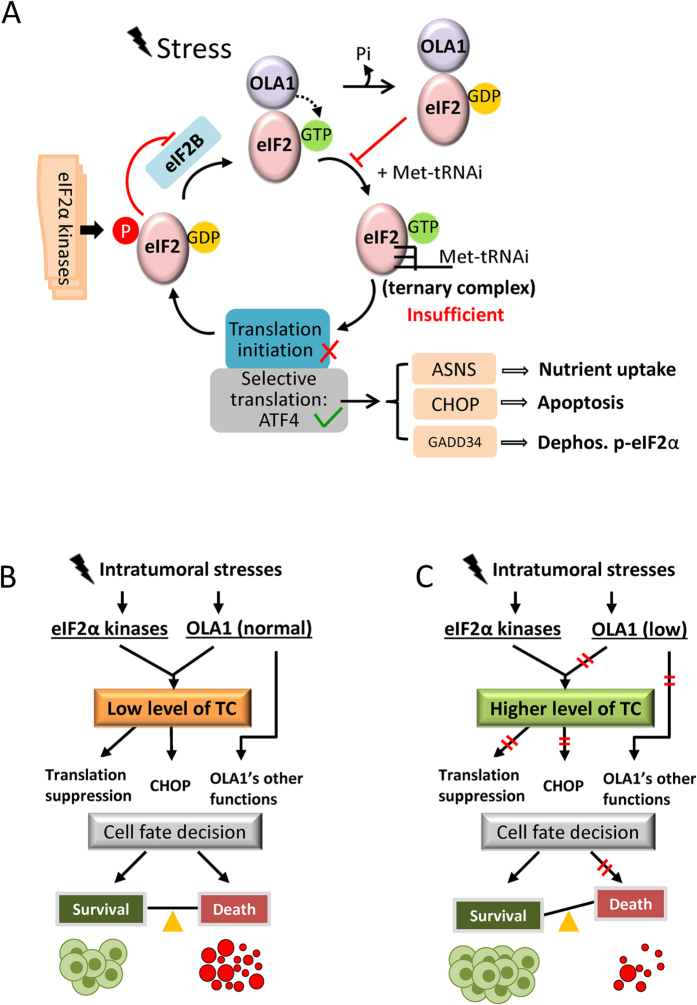
Proposed role of OLA1 in regulating protein synthesis and ISR. (**A**) Model for eIF2-mediated translational control. In addition to the eIF2α phosphorylation mechanism that inhibits the regeneration of eIF2-GTP from eIF2-GDP by eIF2B, we propose that OLA1 directly binds eIF2 and converts eIF2-GTP to eIF2-GDP via its GTPase activity, thus providing a 2^nd^ mechanism that limits the availability of TC. (**B**,**C**) The balance between cancer cell survival vs. death in the presence of OLA1 (**B**) or its deficiency (**C**). Cancer cells within a solid tumor are under multiple chronic stresses and OLA1 has a function in lowering TC level as described in (**A**). When OLA1 is downregulated, a relatively higher level of TC would result in less active ISR-CHOP signaling and tilt the balance towards survival.

The role of OLA1 as a suppressive factor in protein synthesis was first revealed *in vitro* (RRL) and then confirmed *in vivo* (reporter assays and metabolic labeling) (Fig. 1 and S1). In the dual reporter assays, OLA1 exhibited a suppressive effect on cap-dependent translation, however, not on HCV IRES-driven translation. It has been previously reported that the latter mechanism is independent of initiation by eIF2^31^,^32^. Subsequently, OLA1 was found to interact with eIF2 (Fig. 2A–D and S2). A key function of eIF2 is to form the TC that delivers Met-tRNA_i_^Met^ to the 40S ribosome to initiate translation. We demonstrated that OLA1 effectively blocks the formation of TC through its intrinsic GTPase activity (Fig. 2E–G and S3A–C). One possible mechanism that explains the effect of OLA1 on TC formation is the following (Fig. 8A): eIF2 complexed with OLA1 binds GTP; this GTP exchanges freely as noted previously in studies of eIF2-GTP complex^33^; the local GTP released from eIF2 is hydrolyzed to GDP by OLA1; and the local GDP (which could be considered a GDP “cloud” in the vicinity of eIF2) binds to eIF2, and the resulting eIF2-GDP complex is unable to subsequently bind Met-tRNA_i_^Met^. It is known that GDP binds tightly with eIF2 and causes inhibition of TC formation^33^,^34^. However, the OLA1-eIF2 interaction may not be the only mechanism by which OLA1 regulates mRNA translation. OLA1 may also affect translation elongation and termination as indicated in yeast systems^35^,^36^. Because initiation is a rate-limiting step in mRNA translation, the mechanisms of its regulation have long been attractive therapeutic targets for human diseases including cancer. The present study demonstrates the feasibility of directly targeting the formation of the TC for intervention in mRNA translation.

Another critical function of eIF2, the regulation of ISR signaling, is also mediated by the availability of TC. ATF4 is minimally translated under normal conditions because sufficient TC allows translation re-initiation to occur at upstream open reading frame(s) (uORF), excluding the ATF4 ORF. However, under stress, the TC deficiency causes delayed ribosome scanning and increased translation of the ATF4 ORF^37^. The best characterized cause of TC deficiency is the phosphorylation of eIF2α by the eIF2α kinases. Nevertheless, other mechanisms have also been proposed, including a reduced level of Met-tRNAi^38^ and altered activity of eIF2B^39^. Here we present OLA1, a suppressor of *de novo* TC formation rather than its regeneration, as a novel regulator of the ISR. OLA1 represents an intrinsic contributor to the ISR process, and downregulation of OLA1 could lead to increased abundance of TC and a less aggressive ISR (Fig. 3 and S4). While the concentration of TC was not directly measured due to technical difficulties, ATF4 levels were analyzed by IB, as the induction of ATF4 can serve as a surrogate marker for TC deficiency^14^,^40^. However, our cell-free and *in vitro* assays failed to show OLA1’s direct effect on eIF2α phosphorylation or dephosphorylation (Fig. S3D and S3E). OLA1 may function differently from other eIF2-binding proteins—notably, p67, which protects eIF2α from phosphorylation by eIF2 kinases^41^.

Our finding of the occurrence of decreased ISR (Fig. 3) and increased survival (Fig. 4) in the same stressed cells falls within a field of controversy. Whereas induction of ISR by blocking dephosphorylation of eIF2α-P was found to promote survival in some cases of stressed cells^42^,^43^, similar strategies (i.e., stimulating HRI to induce eIF2α-P) were developed instead to limit cancer cell proliferation and tumor growth^14^,^40^. In the latter case, decreased TC formation was proposed as the mechanism of action. In our study, the increased survival of OLA1-KD cells under stresses may be attributable to the increased abundance of TC, as the phenotype could be abolished when the cells were pretreated with TC-depleting agents (Fig. 4C). It is likely that a less severe decrease in TC could maintain the translation of certain proteins that promote cell survival, but delay the expression of ATF4 targets that may trigger apoptosis.

When cultured *in vitro*, OLA1-KD cells proliferated normally or slower than control cells (Fig. S6A–E). To our surprise, when the OLA1-KD breast cancer cells were inoculated *in vivo*, they grew into much larger late-stage tumors with more metastasis (Figs 5 and 6 and S7). This phenotype was in fact the result of a net gain of cells due to decreased apoptosis which is likely caused by hypoactive ISR and largely diminished CHOP (Fig. 5C,D). CHOP is a direct target of ATF4; it triggers apoptosis by repressing the pro-survival gene BCL2 and inducing a network of pro-apoptotic genes^44^. Based on these findings and the aforementioned *in vitro* analyses, we interpret the role of OLA1 in tumor growth as follows (Fig. 8B,C): under intratumoral stresses, interaction of OLA1 with eIF2, which suppresses TC formation, and phosphorylation of eIF2α, which blocks TC regeneration, are co-induced to deplete TC and activate ISR. The outcome of this “dual” constituted ISR is a balance between death and survival. However, with the withdrawal of OLA1, the resulting hypoactive ISR-CHOP signaling tilts the balance in favor of survival. Therefore, OLA1 can be considered an important player in the cell fate decision process for a solid tumor, through its role in, but not limited to, the regulation of ISR. It is noteworthy that more active GSK3β signaling was also detected in OLA1-KD tumors (Fig. 5D). Although the active GSK3β indicates a resting, less stimulated cell, current evidence from the literature is not sufficient to support that hyperactive GSK3β can cause attenuated ISR, or *vice versa*^45^. These two events may result from OLA1’s independent functions.

In orthotopic breast cancer models, OLA1-KD tumors have markedly increased potential to spread to LNs and lungs (Fig. 6). Expression of proteins known to regulate EMT were found unchanged (MTA1, β-catenin, and Vimentin), or even decreased in the KD tumors (Snail) (Fig. 5D). However, the invasiveness of the KD cancer cells was evidenced by a higher incidence of intravasation (Fig. 6). During metastasis, cancer cells must survive a variety of stresses such as anoikis, hemodynamic shear forces, and unfavorable conditions at the remote site^46^. It is possible that the OLA1-KD-mediated increased survival renders cancer cells able to overcome these rate-limiting steps of metastasis. Interestingly, a recent report by Matsuzawa *et al.* demonstrated OLA1’s interaction with BRCA1 and its inhibitory role in centrosome amplification^47^. OLA1-KD cells may thus acquire genetic instability necessary for malignant progression.

Our patient-based study furnished evidence that lower OLA1 expression is associated with a higher chance of cancer relapse and worse DSS (Fig. 7). This is consistent with the fact that lower OLA1 mRNA levels were correlated with decreased overall survival in TCGA breast cancer patients (Fig. S9F). However, in the same dataset, OLA1 mRNA levels appears to be higher in tumors compared to adjacent normal tissues (Fig. S9B). Currently we are not clear about the role of OLA1 in tumorigenesis, nor could our data rule out the requirement of OLA1 in *in vitro* growth and early-phase tumor growth (Fig. 5 and S6). Nevertheless, our IHC analysis of clinical samples provides an evaluation of OLA1 expression at the protein level, and suggests that lower OLA1 may serve as a risk factor of worse outcome, especially for patients undergoing adjuvant chemotherapy. Future studies are warranted to delineate the mechanisms that mediate OLA1 downregulation and cancer relapse — for example, the development of resistance to post-surgery chemotherapy, or a switch between cancer dormancy and recurrence. In conclusion, the present study establishes the role of OLA1, an ancient TRAFAC class *P*-loop GTPase, in the regulation of protein synthesis and stress-induced translational control. By regulating TC formation, and other cellular processes to be further characterized, OLA1 participates in ISR and cell fate decisions in stressed cells, impacting the outcome of stress response in general and specifically, the overall growth and progression of a tumor.

## Materials and Methods

### Cell lines

Cell lines HEK293T (human embryonic kidney), MDA-MB-231 (human breast cancer), HeLa (human cervical cancer), A549 (human lung carcinoma), and HK-2 (human renal proximal tubule) were obtained from the American Type Culture Collection, while the luciferase-expression MDA-MB-231-luc-D3H2LN was from Xenogen (now PerkinElmer). Cells were cultured in Dulbecco’s Modified Eagle’s medium (DMEM, Sigma-Aldrich) containing 10% fetal bovine serum (Thermo Scientific), 100 units/ml penicillin and 100 mg/ml streptomycin (Lonza). Primary mouse embryonic fibroblast (MEF) cells were prepared from 14.5 day embryos harvested from heterozygous-heterozygous crossing of an OLA1 knockout line as described previously^21^.

### Cell transfection and transduction

For transient RNAi, human OLA1 cDNA (NM_013341.3)-specific siRNA (SASI_Hs01_00244684) and the control siRNA (MISSION siRNA Universal Negative Control #1 SIC001) were acquired from Sigma-Aldrich, and the DharmaFECT Transfection Reagents (Thermo Scientific) were used to deliver the siRNA to the cells following the manufacturer’s instructions. To establish stable OLA1-KD cell lines, two approaches were employed. In Approach 1, the pLVTHM plasmid (Addgene, #12247) was engineered to express shRNA for OLA1 (sh-OLA1) or a control non-targeting sequence (sh-control), i.e., 5′-CCGGGAGGAAATGATTGGGCCCATTCTCGAGAATGGGCCCAATCATTTCCTCTTTTTTG-3′ for sh-OLA1 and 5′-CCGGCAACAAGATGAAGAGCACCAACTCGAGTTGGTGCTCTTCATCTTGTTGTTTTTG-3′ for sh-control, and transfected into the parental MDA-MB-231 cells^21^. Cell clones were selected from the transfected population by serial dilution and subcultured for more than a month. Immunoblot analysis was used to verify the efficiency of OLA1-KD in each sub cell line. In Approach 2, SMARTvector lentiviral shRNA particles (Thermo Scientific) containing the OLA1-specific shRNA sequence (5′-TGTTCGCTTCCAGATACTT-3′) or the control shRNA sequence were used to transduce cell cultures at a range of 5–20 TU/cell. Cells expressing the respective shRNAs were selected with puromycin (5 μg/ml) for 1 month. The knockdown efficiency of the target gene was verified by western blot analysis. To achieve ectopic OLA1 expression, cDNA of OLA1 full-length open reading frame (OLA-wt, 396 aa), OLA1-N230A (point mutation at aa 230), or OLA1-ΔTGS (C-terminal deletion of the whole TGS domain, 304 aa) was cloned into the pCMV-Tag1 vector (Stratagene), allowing the expression of N-terminal FLAG-tagged wild-type or mutant OLA1 proteins. Alternatively, OLA1-WT was cloned into the pdEYFP-N1gen plasmid with a C-terminal YFP tag^21^. To reconstitute OLA1 expression in OLA1-KD cells, the above OLA1-WT pCMV-Tag1 was converted to an OLA1-rescue plasmid (FLAG-OLA-res) by modifying nucleotide sequence corresponding to the OLA1-shRNA (5′-AAGTATCTGGAAGCGAACA-3′) into 5′-aaatacctcgaggcaaata-3′ without changing the encoded amino acid sequence. To express tagged proteins for immunoprecipitation, the OLA-WT, OLA1-N230A, and OLA1-ΔTGS cDNAs were cloned into the pIRESneo3-FLAG vector (Clontech). Full length cDNAs encoding wild-type eIF2α and the S51A mutant eIF2α were obtained from Addgene (Cambridge, #21807 and #21808) and re-cloned into a pcDNA3.1 vector to express eIF2α proteins fused with an HA-tag at their C-terminus. HEK293T cells were transfected with these DNA constructs for 48 h before they were harvested for immunoprecipitation. To study *in vivo* translation initiation efficiency, a bicistronic reporter (rLuc-IRES-ffLuc) was constructed with the pRL-CMV plasmid (Promega), in which the HCV 5′-UTR (nucleotides 14–383, Genbank #M62321.1) was placed between the upstream Renilla luciferase (rLuc) gene and the downstream firefly luciferase (ffLuc) gene, and the whole insert was driven by the CMV promoter (Fig. 1C). At 48 h after the co-transfection of an OLA1-expression vector and the bicistronic reporter, or at 72 h after the co-transfection of the siRNA and the reporter, luciferase and Renilla activities were assayed using Promega Bright and Renilla Glo kits.

### Cell-free mRNA translation

The Flexi® Rabbit Reticulocyte Lysate System (Promega) was used to synthesize luciferase protein using the Luciferase Control RNA (Promega) as the template mRNA following the manufacturer’s instructions. In translation inhibition studies, recombinant His-tagged human OLA1-WT protein and its mutant forms (N230A, ΔTGS), the recombinant red fluorescent protein (HIS-RFP), or the purified actin (Sigma), was incubated at concentrations up to 590 nM with the translational reaction mixture at 30 °C for 30 min before the mRNA was added. After incubating at 30 °C for 60 min, the translation was stopped on ice, and the product (luciferase) was measured with the BrightGlo Luciferase Assay System (Promega). All recombinant proteins used in these studies were custom made by Epoch Life Science.

### Metabolic labeling and *in vivo* protein synthesis analysis

Equal numbers of cells were seeded in a 12-well plate and incubated for 24 h. After the indicated treatment, e.g., serum starvation, amino acid starvation, or tunicamycin (2 μg/ml, Sigma), cells were washed twice with DMEM (without methionine and cysteine) containing dialyzed fetal bovine serum and then incubated with [35S] EXPRESS™ Protein Labeling Mix (Perkin Elmer) for the indicated time. After incubation, the cells were scraped into lysis buffer (Cell Signaling Technology), and 5 μl of the cell lysate was subjected to 10% trichloroacetic acid (TCA) precipitation and the pellets were dissolved in Soluene (Perkin Elmer). Ultima Gold scintillation fluid (Perkin Elmer) was added before scintillation counting in a Tri-Carb 2910TR Liquid Scintillation Analyzer (Perkin Elmer). The radiolabeled protein content was used to quantify the rate of *de novo* protein synthesis. Alternatively, equal amounts of cell lysate were subjected to SDS-PAGE gel and the gel was dried and autoradiographed overnight.

### Polysome profiling

Cell lysates of HEK293T and A549 cells for sucrose gradient centrifugation were prepared in polysome extraction buffer (20 mM HEPES at pH 7.6, 100 mM KCl, 5 mM MgCl_2_, 1 mM dithiothreitol [DTT], 100 μg/ml cycloheximide, 100 U/ml RNAse inhibitor [Thermo Scientific] and Halt Protease and Phosphatase Inhibitor Cocktail [Thermo Scientific]). Extracts were incubated on ice for 20 min, and insoluble material was pelleted by centrifugation at 12000 rpm for 10 min. The resulting supernatant extracts were loaded onto a 10–50% (w/v) sucrose gradient and ultracentrifuged at 30000 rpm for 3 hours at 4 °C in a Surespin 630 rotor (Sorvall). Following centrifugation, the gradients were fractionated using a fraction collector (Brandel), and their quality was monitored at 254 nm using a UA-6 Absorbance Detector (Isco). Proteins from sucrose gradient fractions were precipitated with 10% (v/v) TCA, separated by SDS-PAGE, and transferred to PVDF membranes for immunoblotting.

### Ternary complex formation

Purified eIF2 (up to 150 nM) was incubated with GTP (1 mM) or GDPNP (0.5 mM, Sigma-Aldrich) at 37 °C for 5 min in reaction buffer (25 mM HEPES-KOH, 80 mM K_2_OAc at pH 7.5, 2.5 mM MgOAc, 1 mM DTT, 2.5% glycerol, and 0.1 μg/μl creatine kinase), then mixed with 2 pmol [^14^C]-labeled yeast initiator tRNAi^Met^ (tRNA Probes Inc.) for 15 min. The reaction was stopped by adding ice-cold wash buffer (40 mM Tris-HCl at pH 7.5, 100 mM KCl, 50 mM MgOAc) and then filtered through a nitrocellulose membrane (Millipore). After being thoroughly washed and air dried, the membrane was subjected to liquid scintillation counting. The radioactive counts were used to quantitate TC formation. OLA1 protein (WT, N230A, and ΔTGS) or actin was added before the addition of [^14^C]Met-tRNA^Met^i, or after the 15 min binding reaction was completed.

### Nucleotide hydrolysis assay

OLA1 protein (1 μM) was incubated with [γ-^32^P]GTP (6000 Ci/mmol, PerkinElmer) or [γ-^32^P]ATP (3000 Ci/mmol) in the presence of 100 μM of the corresponding unlabeled nucleotides in a 50 μl reaction buffer (25 mM HEPES at pH 7.5, 80 mM K_2_OAc, 2.5 mM MgOAc, and 1 mM DTT) at 35 °C. At various time points, the reaction was stopped by the addition of 200 μl of activated charcoal suspension (100 mg/ml charcoal (Sigma Aldrich) in 1 N HCL). After two centrifugations at 100000 × *g*, release of [^32^P] phosphate in the supernatant was measured by scintillation counting.

### Mouse models of breast cancer growth and metastasis

The animal experiments were carried out in accordance with NIH guidelines and were approved by the Institutional Animal Care and Use Committee at the Houston Methodist Research Institute. To grow xenograft tumors, MDA-MB-231-derived OLA1-KD (shOLA1) and control (shCTL) stable cell lines were injected into left and right shoulders, bilaterally, of 6 week old female nude mice (Charles River Laboratories). Tumors were measured every day for 2 wk. To induce the orthotopic breast cancer model, MDA-MB-231 (D3H2LN)-derived shOLA1 and shCTL cells were injected into the 4^th^ fat pads of nude mice from Charles River or FoxChase SCID from Harlan (all age 6–8 wk). Parameters of tumor growth were acquired twice a week. To monitor possible remote site metastasis in the SCID mice, *in vivo* BLI was taken once a week using IVIS™ 200 (PerkinElmer).

### Patients and specimens

The protocol of human subject application was reviewed and approved by the Human Subject Review Committee of the Second Affiliated Hospital of Zhejiang University, College of Medicine (China). The methods were carried out in accordance with the approved guidelines. Informed consent was obtained from all patients. A total of 160 cases of breast cancer specimens were collected from the hospital. All patients were followed after the surgery, with a median follow-up period of 69 months (range 9–103 months). ANOVA was used to evaluate the relationship between the expression of OLA1 and some pathologic features. Kaplan-Meier survival analysis (log-rank test) was used to evaluate the relationship between OLA1 expression and the relapse risk as well as DSS. SPSS Version 13.0 software (SPSS Inc., Chicago, IL) was used for all statistical analyses. P < 0.05 was considered to be statistically significant.

## Additional Information

**How to cite this article**: Chen, H. *et al.* OLA1 regulates protein synthesis and integrated stress response by inhibiting eIF2 ternary complex formation. *Sci. Rep.* **5**, 13241; doi: 10.1038/srep13241 (2015).

## Supplementary Material

Supplementary Information
