# Supplementary material for: OLA1 regulates protein synthesis and integrated stress response by inhibiting eIF2 ternary complex formation
Source: Sci Rep. 2015 Aug 18;5:13241. doi: 10.1038/srep13241 (PMC4539610; doi:10.1038/srep13241)
Supplement: Supplementary Information [file srep13241-s1.pdf]

Supplementary Information

OLA1 regulates protein synthesis and integrated stress response by inhibiting eIF2 ternary complex formation

Huarong Chen,[1,2#] Renduo Song,[1#] Guohui Wang,[1#] Zonghui Ding,[1] Chunying Yang,[3] Jiawei Zhang,[2] Zihua Zeng,[4] Valentina Rubio,[1] Luchang Wang,[1] Nancy Zu,[1] Amanda M. Weiskoff,[1] Laurie J. Minze,[5] Prince V.S. Jeyabal,[1] Oula C. Mansour,[1] Li Bai,[1] William C. Merrick,[6] Shu Zheng,[2†] and Zheng-Zheng Shi [1†]

[1]Department of Translational Imaging

[3]Department of Radiation Oncology

[4]Department of Pathology and Genomic Medicine

[5]Immunobiology Research Center

Houston Methodist Research Institute, Weill Cornell Medical College, Houston, TX 77030, USA

[2]Cancer Institute, The Second Affiliated Hospital, School of Medicine, Zhejiang University, Hangzhou, Zhejiang 310009, China

[6]Biochemstry Department, Case Western Reserve University, Cleveland, OH44106, USA

[#]These authors contributed equally to this work

[†]Corresponding authors

Supplementary Figures

A

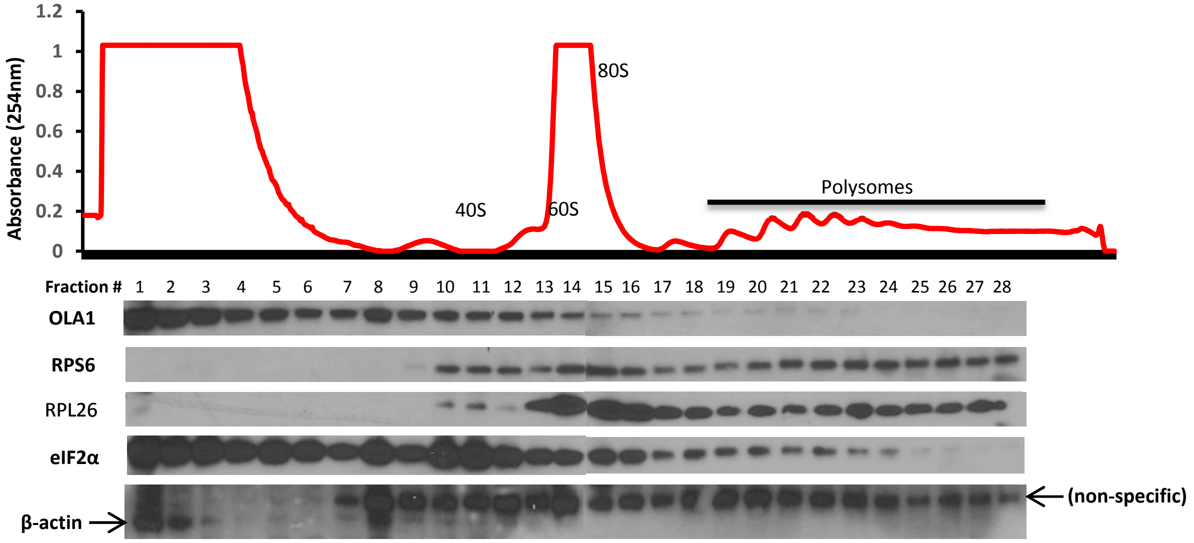

B

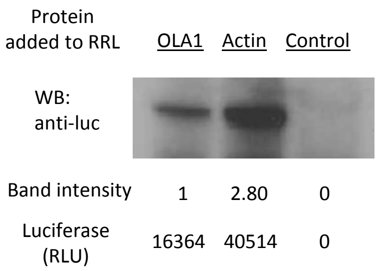

C

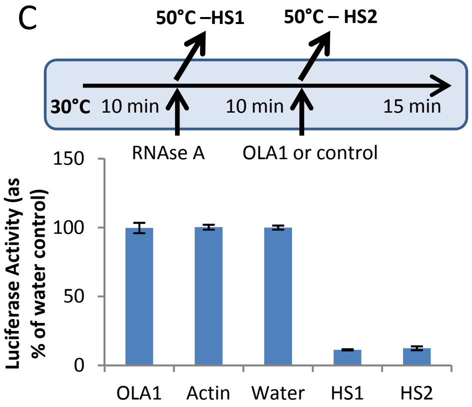

D

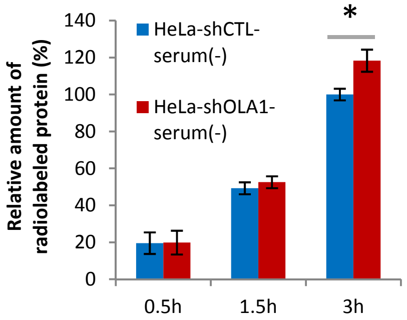

E

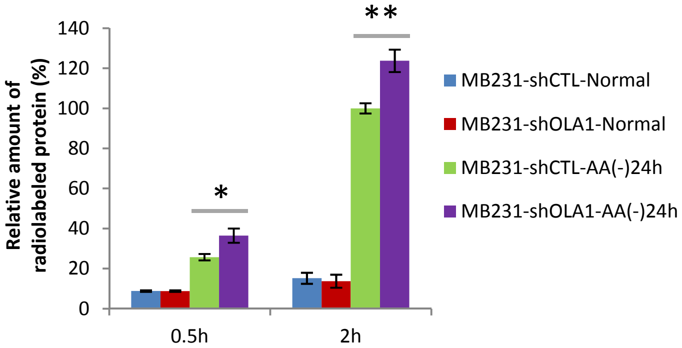

F

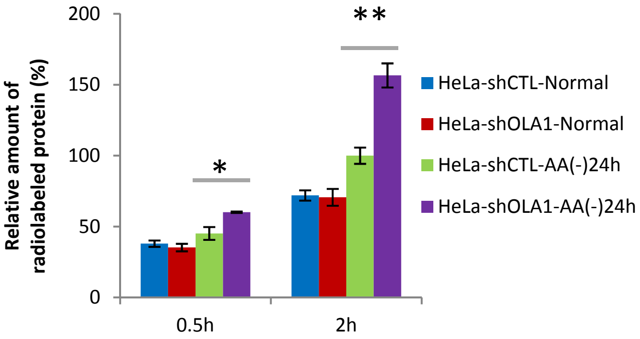

Supplementary Figure 1. OLA1 suppresses mammalian protein synthesis. (A) Distribution of OLA1 in 10-50% sucrose density gradient fractions of HEK293T cell extract as measured by IB. Distributions of rpS6 (a small subunit protein) and rpL26 (a large subunit protein) are shown for comparison. β-actin was used as negative controls. Positions of the S40, S60, S80 and polysome fractions are also indicated. **(B)** Effect of OLA1 on luciferase protein synthesis in a Rabbit Reticulocyte Lysate (RRL) system as compared with actin. Luciferase levels as measured by luminescence (RLU) corresponded to amount of protein detected on immunoblot. Control: no template mRNA. **(C)** OLA1 has no effect on synthesized luciferase protein. Luciferase protein synthesis in a RRL system was stopped with RNAse A 10 min after the start of incubation. After 10 min incubation with RNAse A, 422 nM OLA1, actin, or an equal volume of H_2O were added. Fifteen minutes later, reactions were halted on ice and measured with BrightGlo Luciferase Assay System. Heat shock treatments at 50°C were performed as a positive control. (n=3). **(D)** Labeling of *de novo* protein synthesis in OLA1-KD HeLa cells. After 24h serum starvation, serum was restored to the shCTL and shOLA1 cells for the indicated times in the presence of the [^{35}S] EXPRESS ™ Protein Labeling Mix. The radiolabeled protein content was used to quantify the protein translation rate. Error bars represent the standard deviation of at least triplicate samples. **(E, F)** Analysis of *de novo* protein synthesis in cancer cells stimulated by amino acid restoration. After 24h methionine and cysteine starvation, shCTL and shOLA1 cells from the MDA-MB-231 origin **(D)** and those from the HeLa origin **(E)** were incubated with the Labeling Mix containing [^{35}S]Met/Cys for the indicated time. All data are shown as mean ± SD. (n =5). Student's t test, * $p < 0.05$; ** $p < 0.01$, NS, not significant.

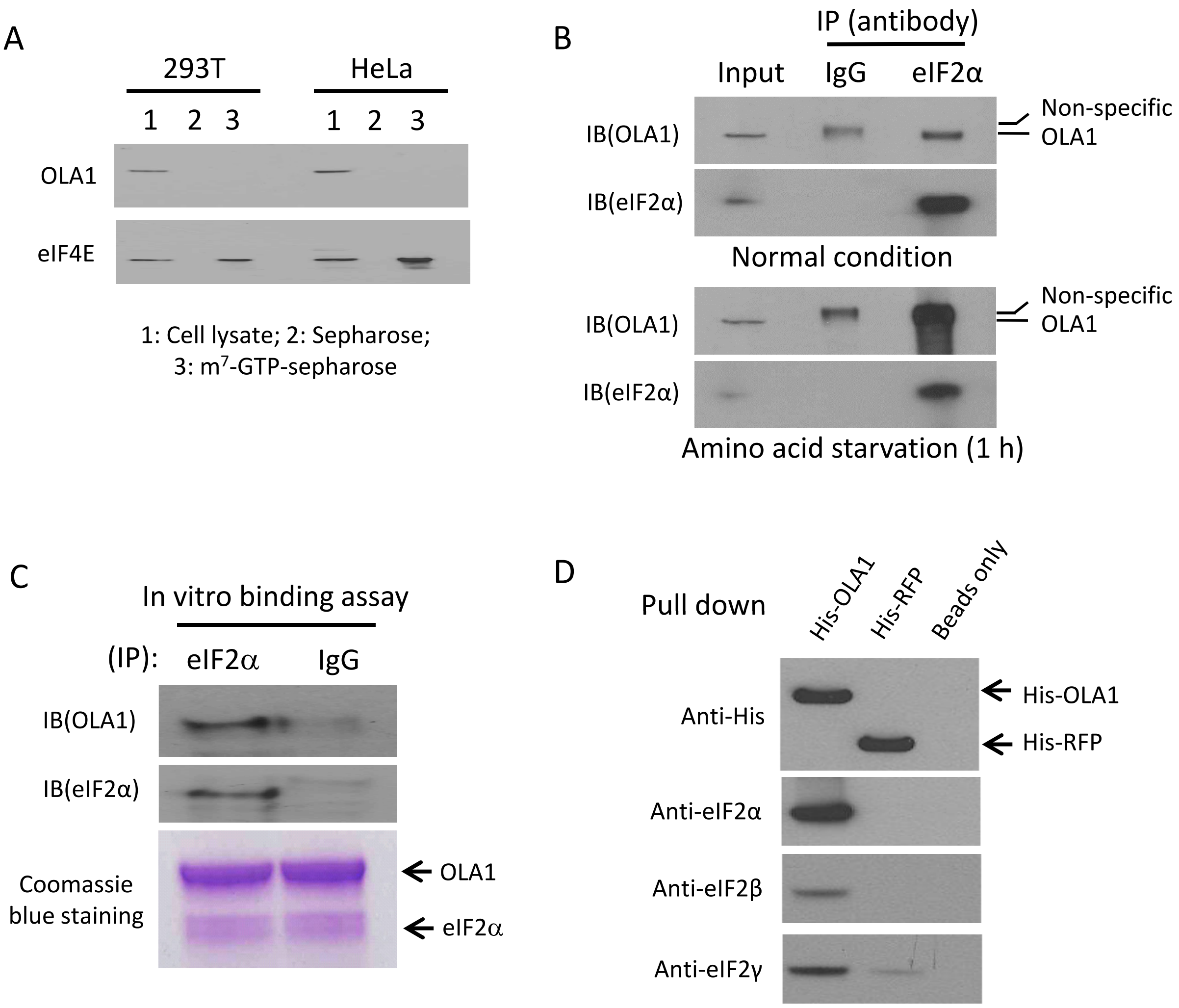

Supplementary Figure 2. OLA1 binds to eIF2. (A) OLA1 does not associate with the cap-binding complex as assessed by the m^7-GTP-sepharose pull-down assay. Total cell lysates prepared from HEK-293T or HeLa cells were incubated with m^7-GTP-sepharose 4B beads or control sepharose beads, and the pull-down products were immunoblotted with anti-OLA1 and anti-eIF4E antibodies. The successful pull-down of eIF4E validated the assay. (**B**) The enhanced binding of endogenously expressed OLA1 and eIF2α under amino acid starvation. HEK-293T cells were cultured in complete medium or the Krebs-Ringer Bicarbonate Buffer for 1 h and the cell lysates were immunoprecipitated with anti-eIF2α antibody or a control antibody (IgG) and the precipitates were immunoblotted with anti-OLA1 and anti-eIF2α antibody. Note that a non-specific protein was present with the IgG IP with a molecular weight slightly larger than OLA1. **(C)** OLA1 binds eIF2α *in vitro*. Recombinant human HIS-eIF2α and OLA1 proteins were co-incubated and immunoprecipitated with anti-eIF2α antibody. The precipitates were then analyzed by immunoblotting with anti-OLA1 and anti-eIF2α antibody (upper panel). The 'input' OLA1 and eIF2α proteins were verified by Coomassie blue staining (lower panel). **(D)** OLA1 binds eIF2 *in vitro*. Purified eIF2 was incubated with recombinant His-tagged OLA1 or the control His-tagged RFP (red fluorescent protein), and the *in vitro* pull-down assay was done with the His-Tag Isolation & Pulldown beads. The pull-down products were immunoblotted with anti-His, eIF2α, eIF2β and eIF2γ antibodies sequentially.

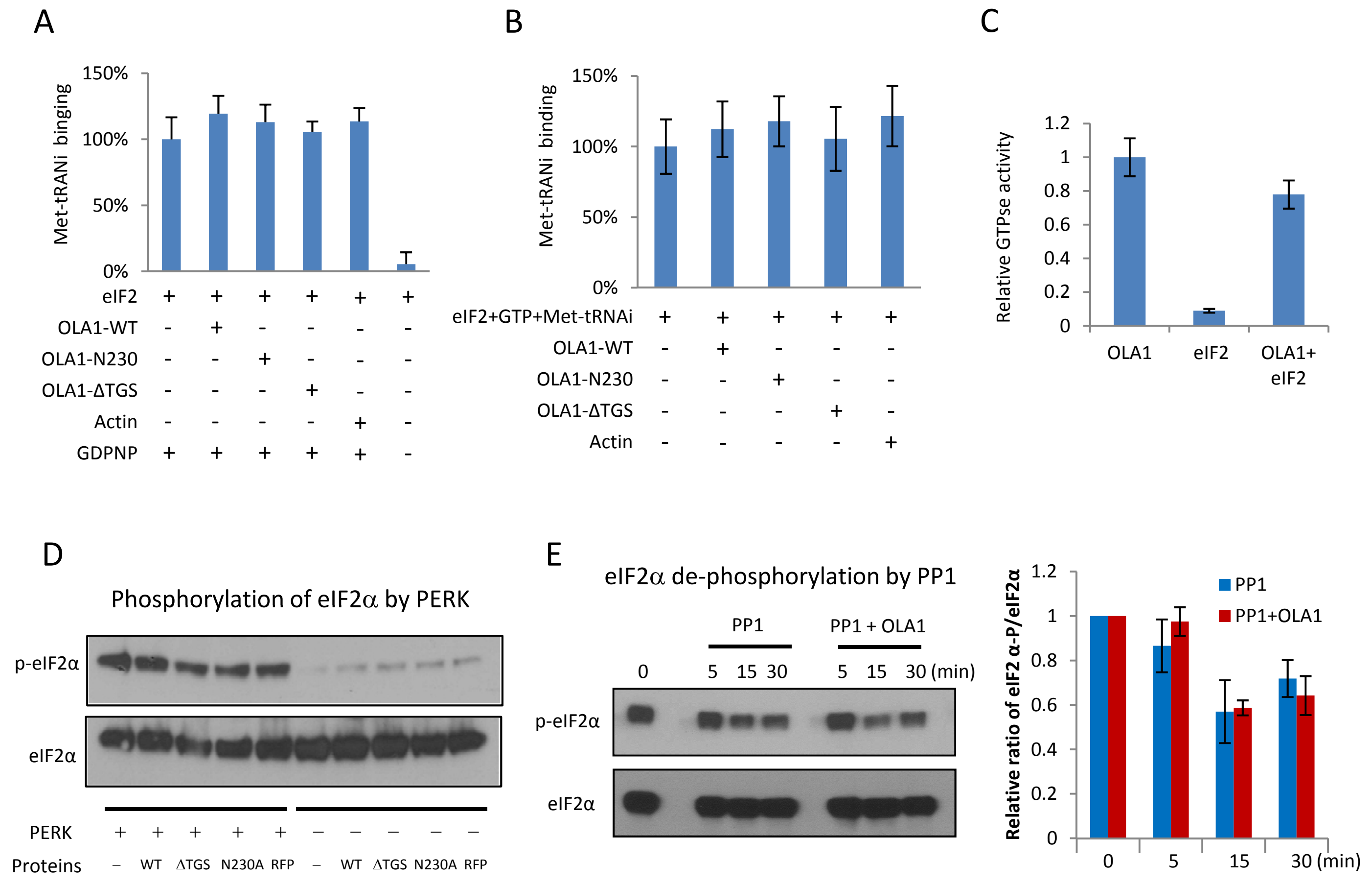

Supplementary Figure 3. OLA1 interferes with the *de novo* formation of TC via its GTPase. (A) Effect of OLA1 on TC formation when GTP was replaced with GDPNP (the non-hydrolyzable GTP analogue). OLA1 protein (OLA1-WT, OLA1-N230A, or ΔTGS) was incubated with purified eIF2 in the presence of the GDPNP. None of the OLA1 proteins (or actin) had an effect on eIF2 binding to Met-tRNAi. (Protein concentration = 150nM). **(B)** The effect of OLA1 on TC post-formation. The TC formation reaction (eIF2+GTP+Met-tRNAi, 15 min) was allowed to complete, followed by addition of OLA1 proteins and another 15 min incubation. (Protein concentration = 150nM). **(C)** OLA1 and eIF2 are not GTPase activating proteins (GAP) to each other. OLA1 and eIF2 proteins were measured alone or together for their activities in hydrolyzing [γ -^{32}P]GTP. **(D)** Effect of OLA1 on eIF2α phosphorylation *in vitro*. Phosphorylation of the eIF2α present within the rabbit reticulocyte lysate was evaluated with immunoblotting analysis with an anti-eIF2α antibody that cross-reacts with the rabbit protein. The assay shows that none of the OLA1 proteins (OLA1-WT, OLA1-N230A, or ΔTGS), or the control protein RFP, could phosphorylate eIF2α or affect the ability of added PERK to phosphorylate eIF2α. (Protein concentration = 400nM). **(E)** Effect of OLA1 on *in vitro* dephosphorylation of eIF2α by PP1. Heavily phosphorylated eIF2α was prepared by incubating purified eIF2 with PKR. De-phosphorylation of p-eIF2α was mediated by addition of recombinant PP1 in the presence or absence of OLA1 for the indicated time. The p-eIF2α and total eIF2α were detected by immunoblotting and quantified by densitometry analysis (inset on the right).

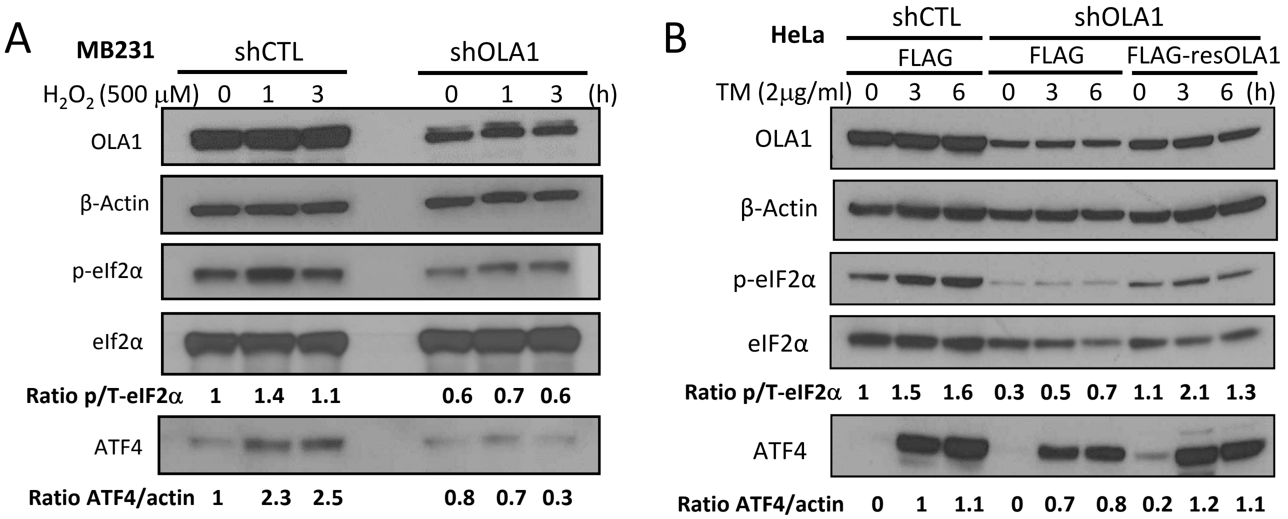

Supplementary Figure 4. OLA1 regulates ISR signaling in cancer cells. (A) MDA-MB-231 shCTL and shOLA1 cells were treated with 500 μM H_2O_2 for the indicated time and analyzed by WB. **(B)** The shCTL and shOLA1 cells of HeLa origin were transfected with Flag-only or FLAG-resOLA1 plasmids for 48h, then treated with 2 μg/ml TM. Cells were subjected to immunoblotting with the indicated antibodies with β-actin used as the loading control.

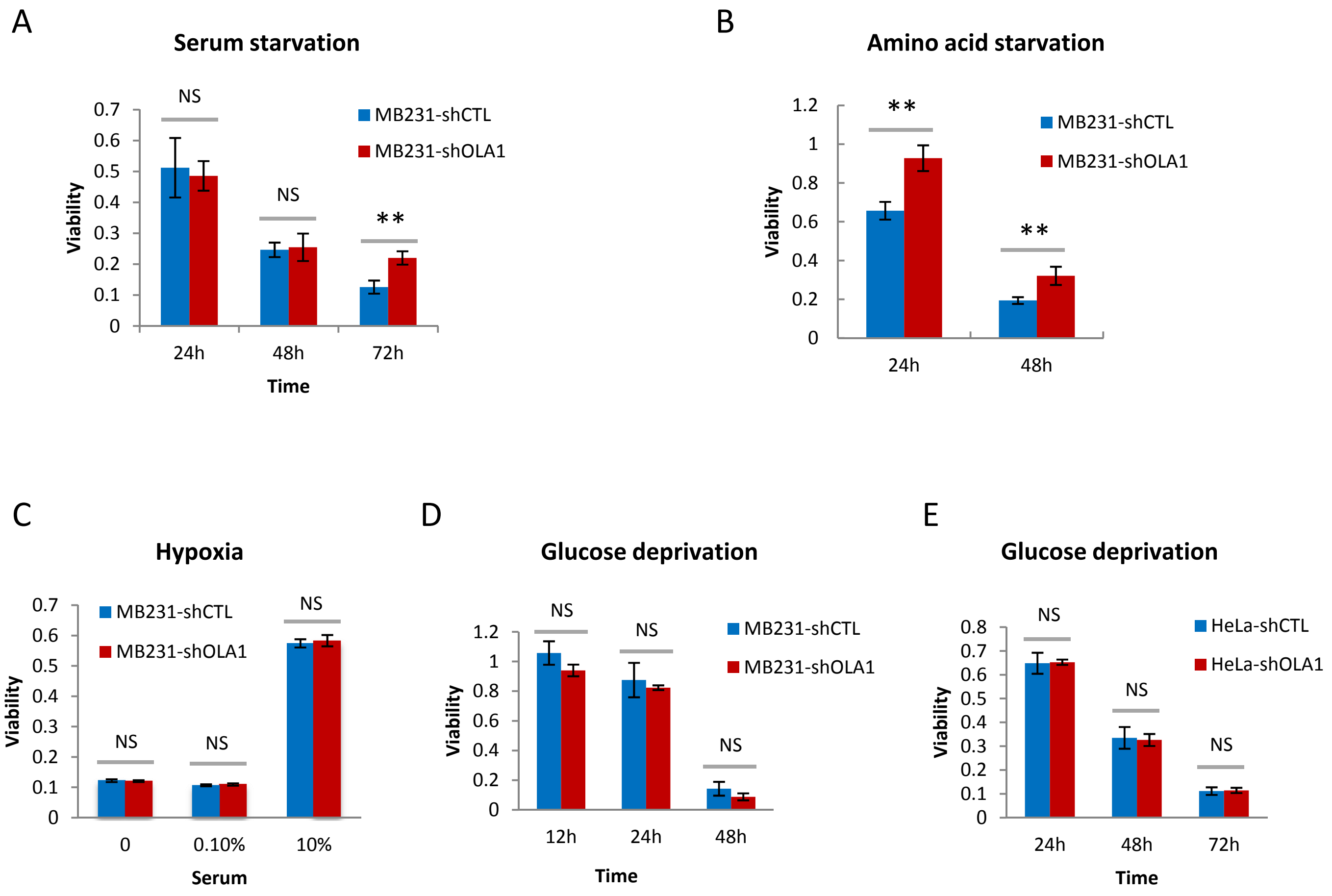

Supplementary Figure 5. (A,B) Susceptibility of MDA-MB-231 shCTL and shOLA1 cells to serum starvation **(A)** and amino acid starvation **(B)**. Cells seeded in normal medium were changed into serum free medium (DMEM) or amino acid-free medium (Krebs-Ringer Bicarbonate Buffer). At the indicated time, cell viability was measured by MTS. Relative viability was normalized against cells cultured in normal medium. **(C)** Susceptibility of MDA-MB-231 shCTL and shOLA1 cells to hypoxia. Cells cultured in DMEM medium supplemented with 0%, 0.1% or 10% FBS were placed into a hypoxia incubator with 1% oxygen for 48h. Relative cell viability was normalized against non-treated cells. **(D, E)** Susceptibility of shCTL and shOLA1 cells of MDA-MB-231 origin **(D)** and those of HeLa origin **(E)** to glucose deprivation. Cells were treated with glucose-free medium and measured for viability at the indicated time. Relative viability was normalized against non-treated cells. Error bars: SD, Student's t test, * $p < 0.05$; ** $p < 0.01$, NS, not significant.

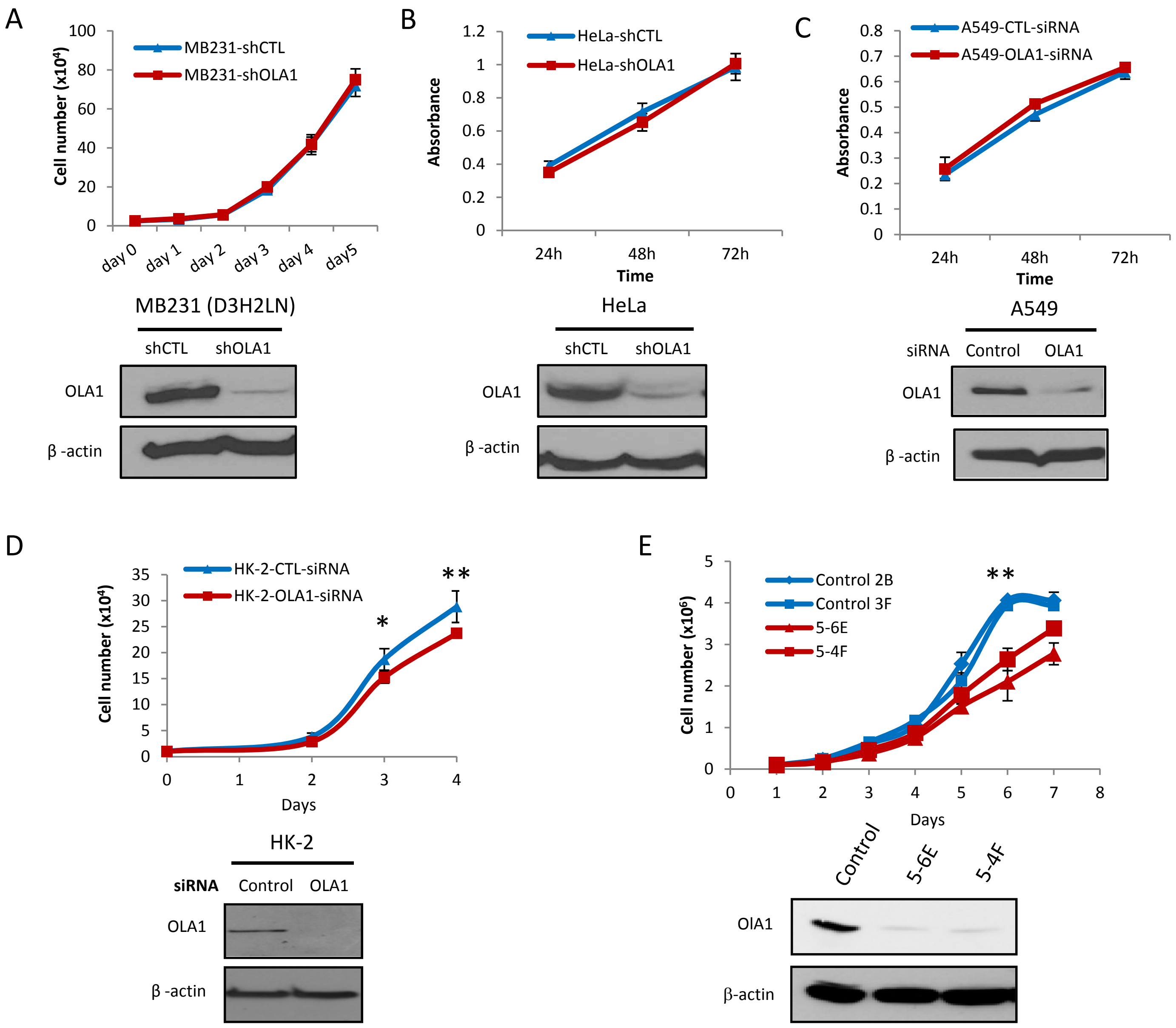

Supplementary Figure 6. Downregulation of OLA1 has no or a negative impact on cell growth *in vitro*. (A) Growth curves of MDA-MB-231 cells stably expressing shCTL or shOLA1 (Approach 2). Cell numbers were obtained by simple cell counting. **(B)** Cell proliferation of HeLa shCTL and shOLA1 cells (Approach 2) was evaluated by MTS absorbance (A490) on 1-3 d after seeding. **(C)** A549 cells were transiently transfected with control or OLA1-specific siRNA and the cell proliferation was evaluated by MTS assay on 1-3 d after the transfection. **(D)** HK-2 cells were transiently transfected with control or OLA1-specific siRNA and the cell proliferation was evaluated by MTS assay on 0-4 d after the transfection. **(E)** MDA-MB-231 cells were stably transfected with control or OLA1 shRNA and cloned into sub cell lines (Approach 1). Growth curves for 2 control and 2 OLA1-KD lines (5-6E and 5-4F) were obtained with simple cell counting. In all these 5 experiments the effectiveness of OLA1-KD was verified by immunoblotting analysis with representative blots shown as insets at the bottom. Where shown, data are presented as means ± SD with indicated significance (* $p < 0.05$; ** $p < 0.01$).

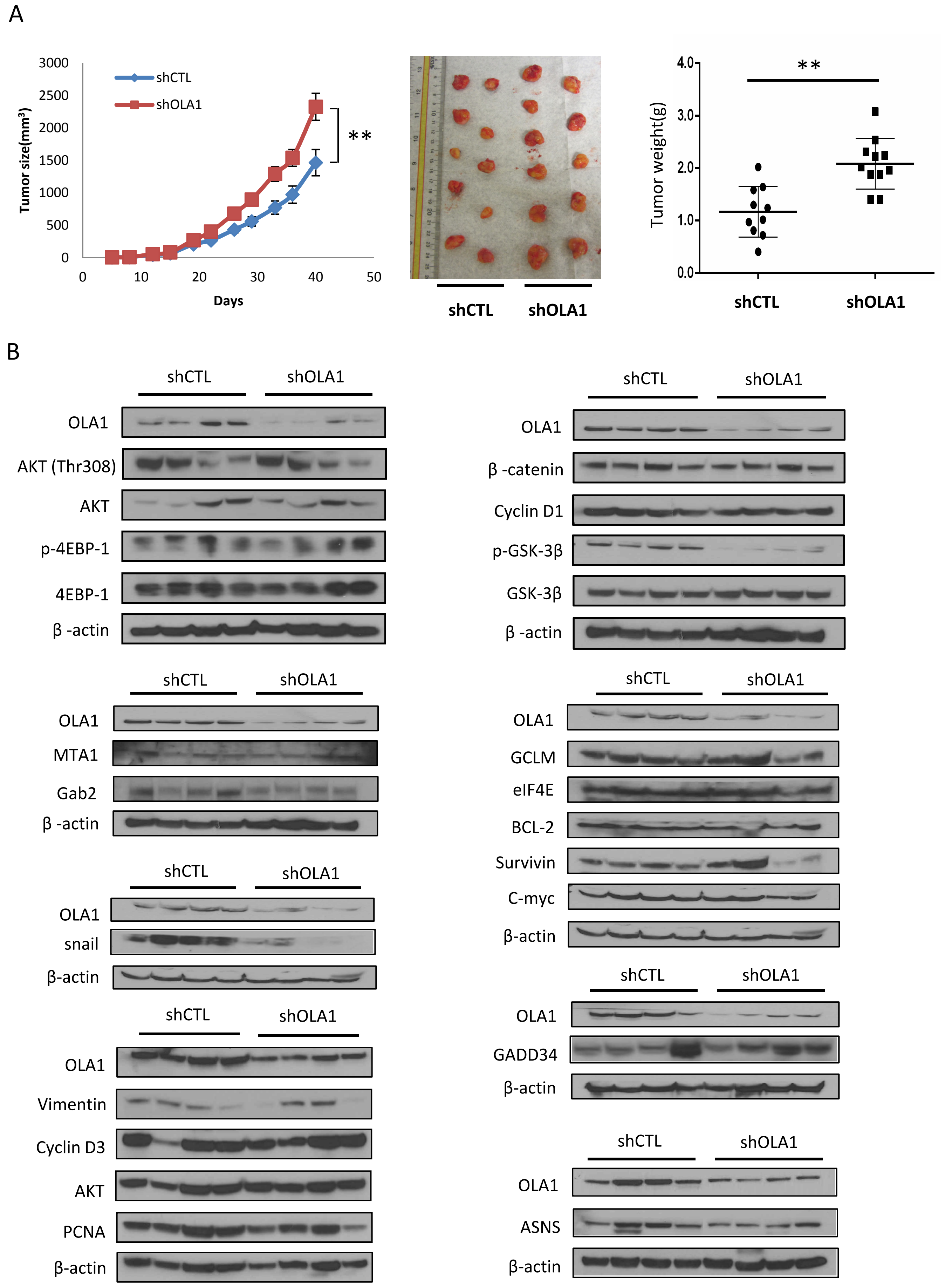
A
3000
2500
2000
1500
1000
500
0
Tumor size(mm³)
shCTL
shOLA1
**
0
10
20
30
40
50
Days
shCTL
shOLA1
4.0
3.0
2.0
1.0
0.0
Tumor weight(g)
**
shCTL
shOLA1
B
shCTL
shOLA1
OLA1
AKT (Thr308)
AKT
p-4EBP-1
4EBP-1
β -actin
shCTL
shOLA1
OLA1
β -catenin
Cyclin D1
p-GSK-3β
GSK-3β
β -actin
shCTL
shOLA1
OLA1
MTA1
Gab2
β -actin
shCTL
shOLA1
OLA1
GCLM
eIF4E
BCL-2
Survivin
C-myc
β-actin
shCTL
shOLA1
OLA1
snail
β-actin
shCTL
shOLA1
OLA1
GADD34
β-actin
shCTL
shOLA1
OLA1
Vimentin
Cyclin D3
AKT
PCNA
β-actin
shCTL
shOLA1
OLA1
ASNS
β-actin

Supplementary Figure 7. Downregulation of OLA1 promotes tumor growth *in vivo*. (A) Growth of xenograft tumors derived from MDA-MB-231 (D3H2LN) cells stably transfected with Control shRNA or OLA1 shRNA (Approach 2). Nude mice were inoculated with the shCTL or shOLA1 cells at their mammary fat pads, and the tumor growth was monitored by measuring tumor sizes (left). All harvested tumors were pictured (middle) and weighted (right). The results are displayed as mean ± SEM (n = 10/11 for shCTL/shOLA1 groups; ** $p < 0.01$; two-way ANOVA test). **(B)** Immunoblotting analysis of tumor tissues harvested from the orthotopic breast cancer model (Approach 2, SCID). The protein extracts were immunoblotted with the indicated antibodies. For each blot OLA1 was probed for verification of the knockdown event and β-actin probed for loading control. Quantitative analyses of these blots resulted in the bar graphs shown in **Figure 5D**.

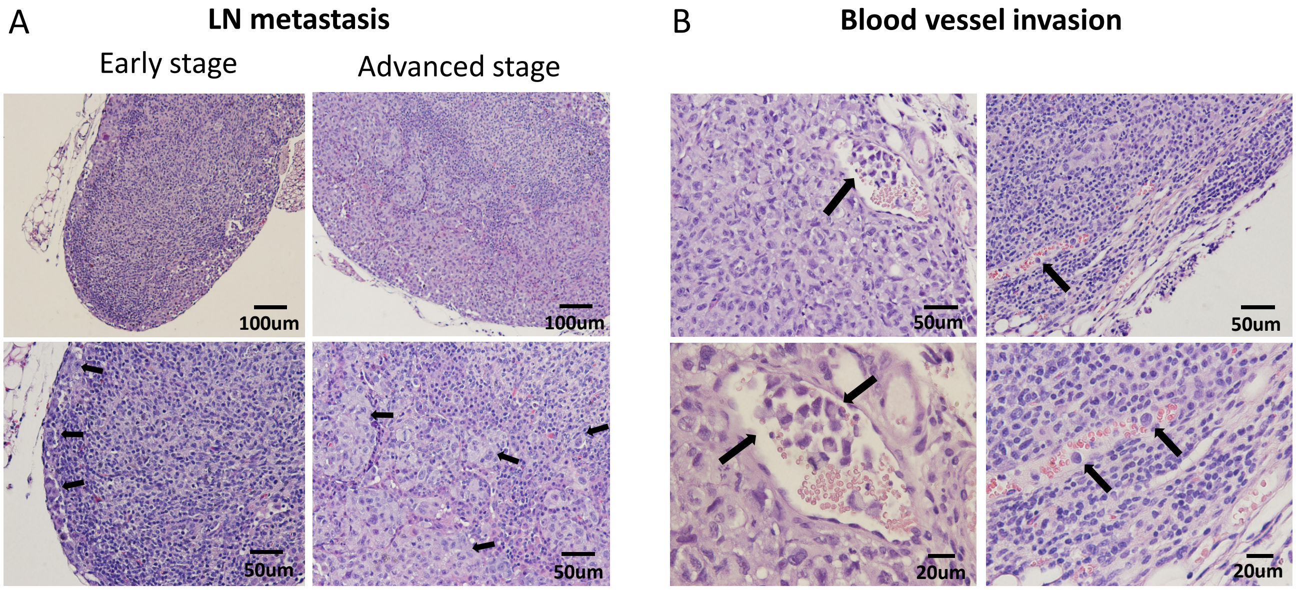

Supplementary Figure 8. Histological assessment of tumor metastasis by H&E staining. (A) Early or late stage of lymph node (LN) metastasis. Example images for each stage are shown with low (upper) and high (lower) magnifications. The arrows indicate metastatic tumor cells. The stage was distinguished by a pathologist considering the invasion depth of tumor cells. (**B**) One example of blood vessel invasion.

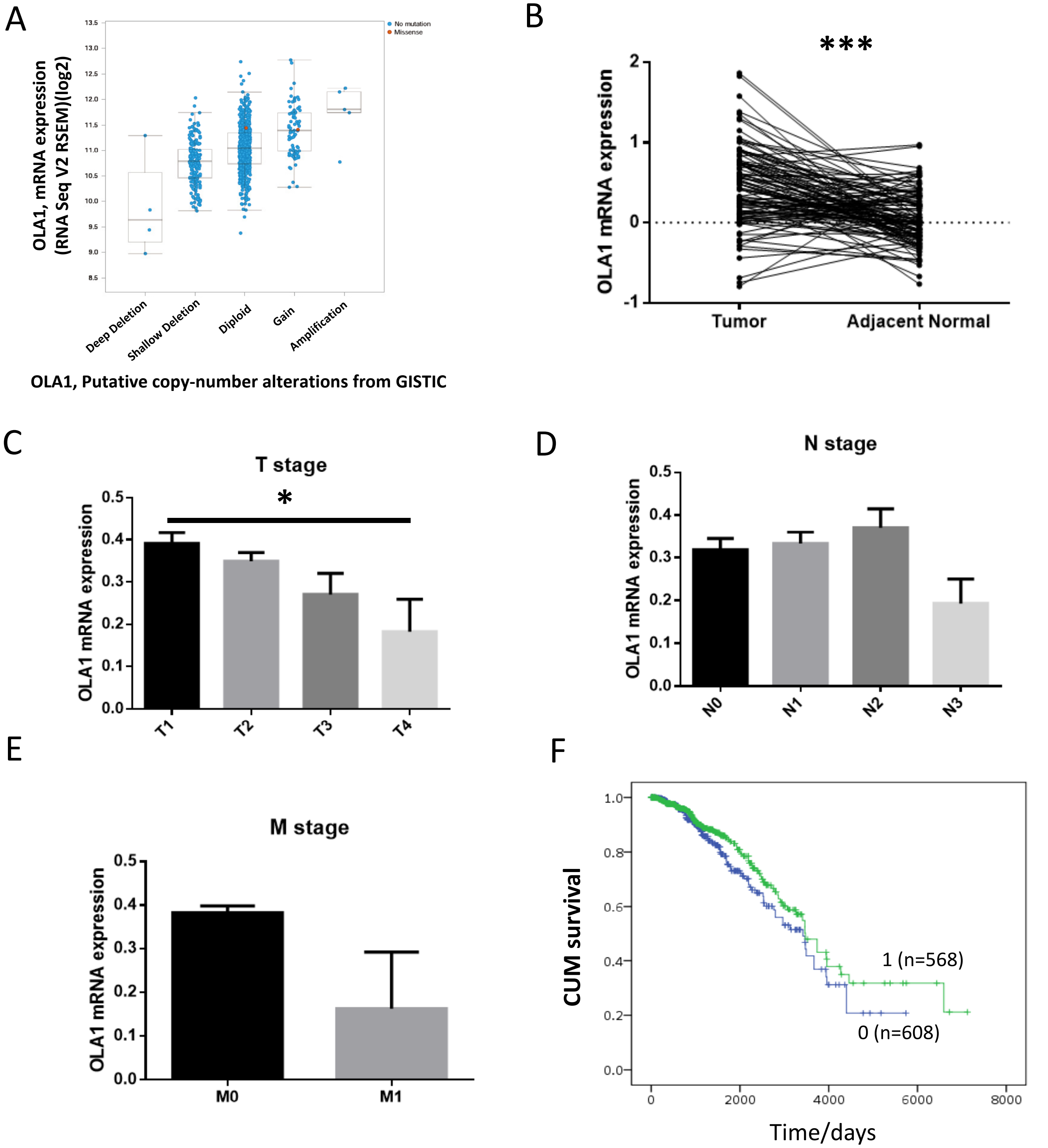

Supplementary Figure 9. Correlation analysis between OLA1 expression and pathoclinical characteristics in breast cancer patients from TCGA cohort (breast invasive carcinoma). (A) Analyses of OLA1 copy number variations and the corresponding mRNA expression changes in a set of 962 case breast cancer patients by The cBio Cancer Genomics Portal [1,2]. **(B)** OLA1 mRNA expression changes between 113 pairs of tumors and adjacent normal tissues. The results are displayed as mean ± SEM. (*** $p < 0.001$; paired-t test). **(C-E)** OLA1 mRNA levels at different TNM stages (T for Tumor, N for Node and M for Metastasis). The results are displayed as mean ± SEM (For **C** and **D**, two-way ANOVA test was used, * $p < 0.05$; For **E**, unpaired-t test was tested). **(F)** Kaplan-Meier analysis for overall survival. OLA1 mRNA level was scored (0 for low expression and 1 for high expression) based on its mean value in all breast cancer patients. p=0.103.

Figure 1A

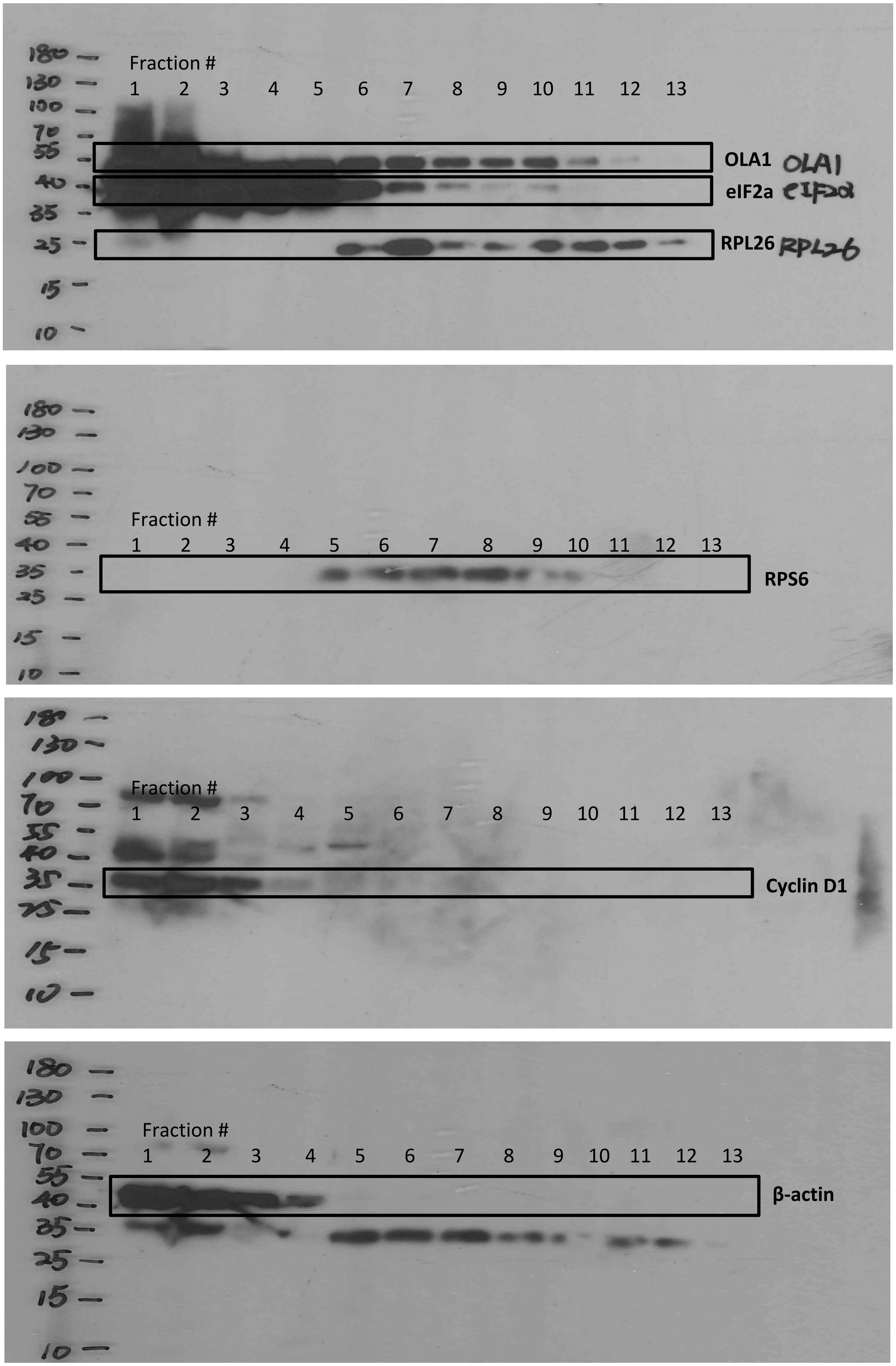

Supplementary Figure 10. Full scan images of immunoblots presented in Figure 1A.

Figure 2B

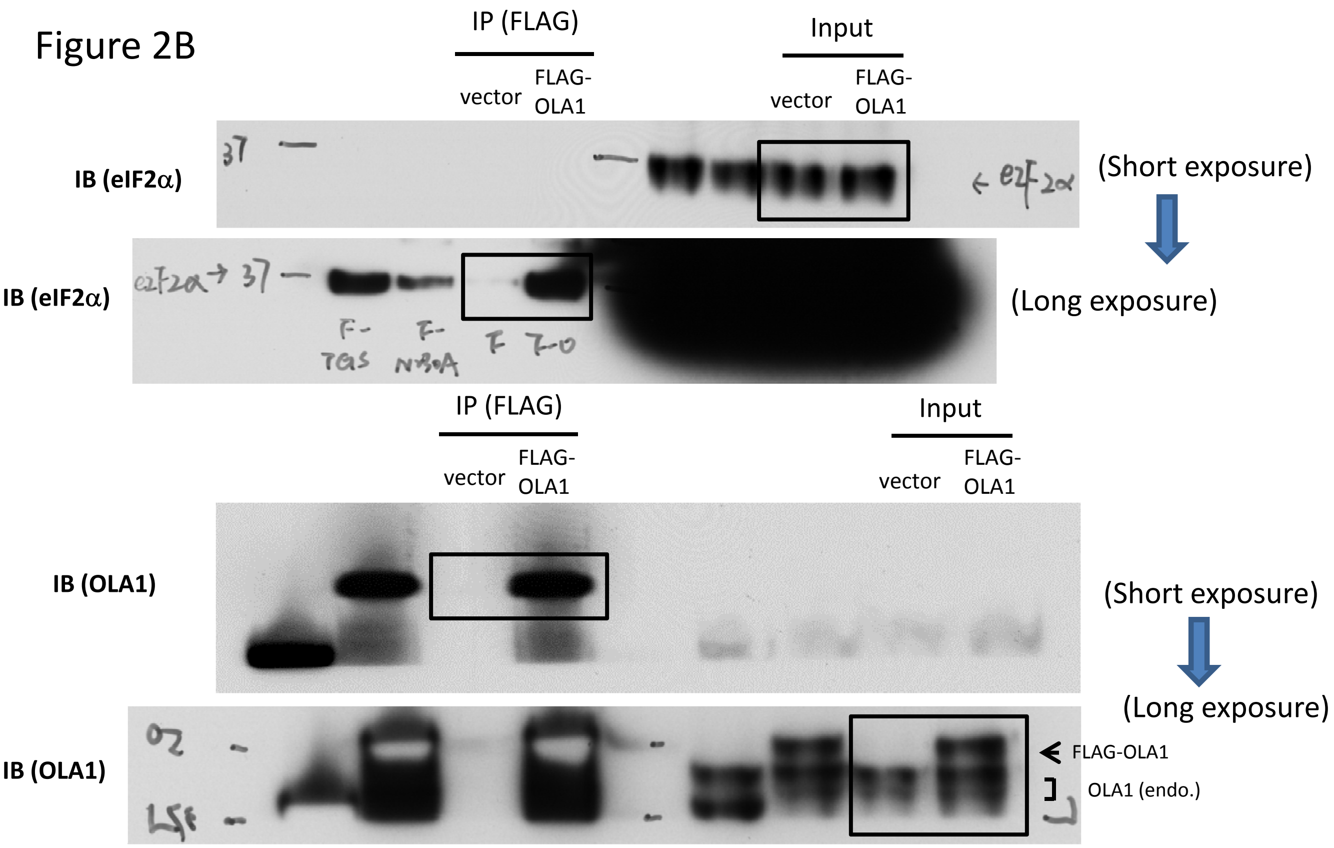

Figure 2C

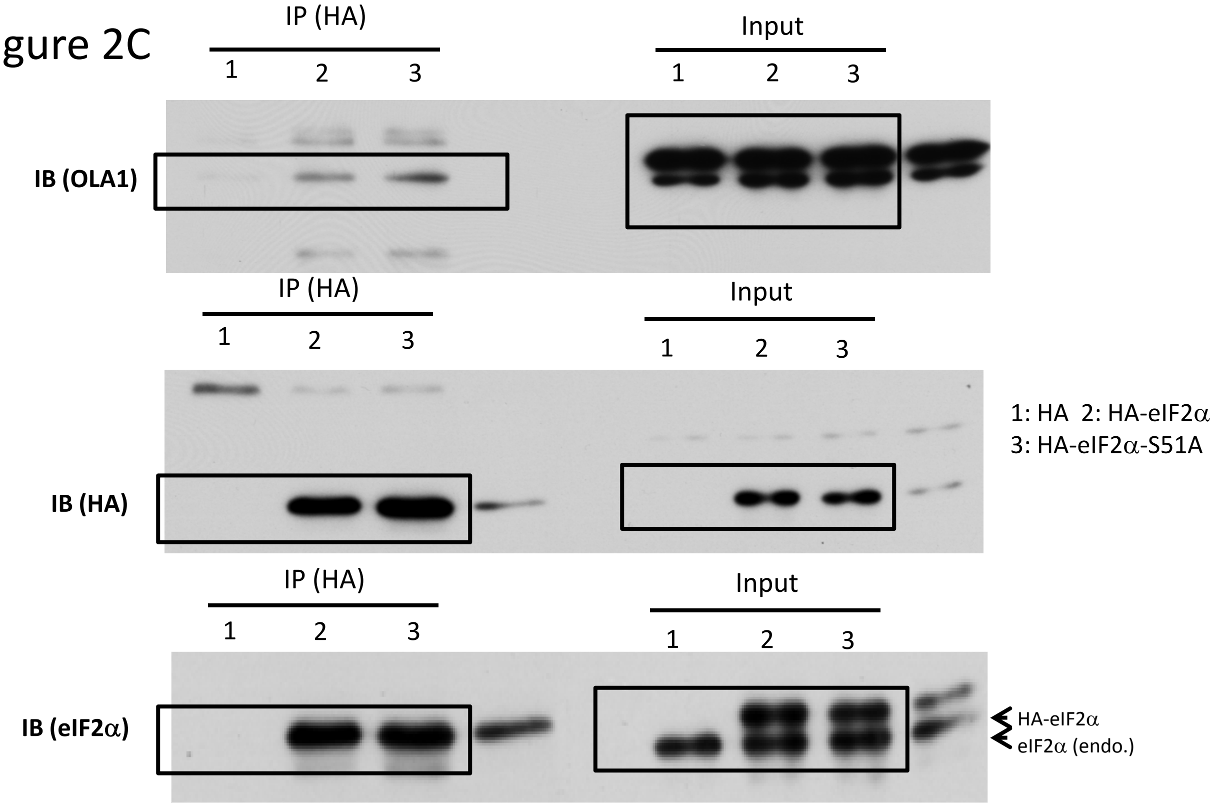

Supplementary Figure 11. Full scan images of immunoblots presented in Figures 2B and 2C.

Figure 3A

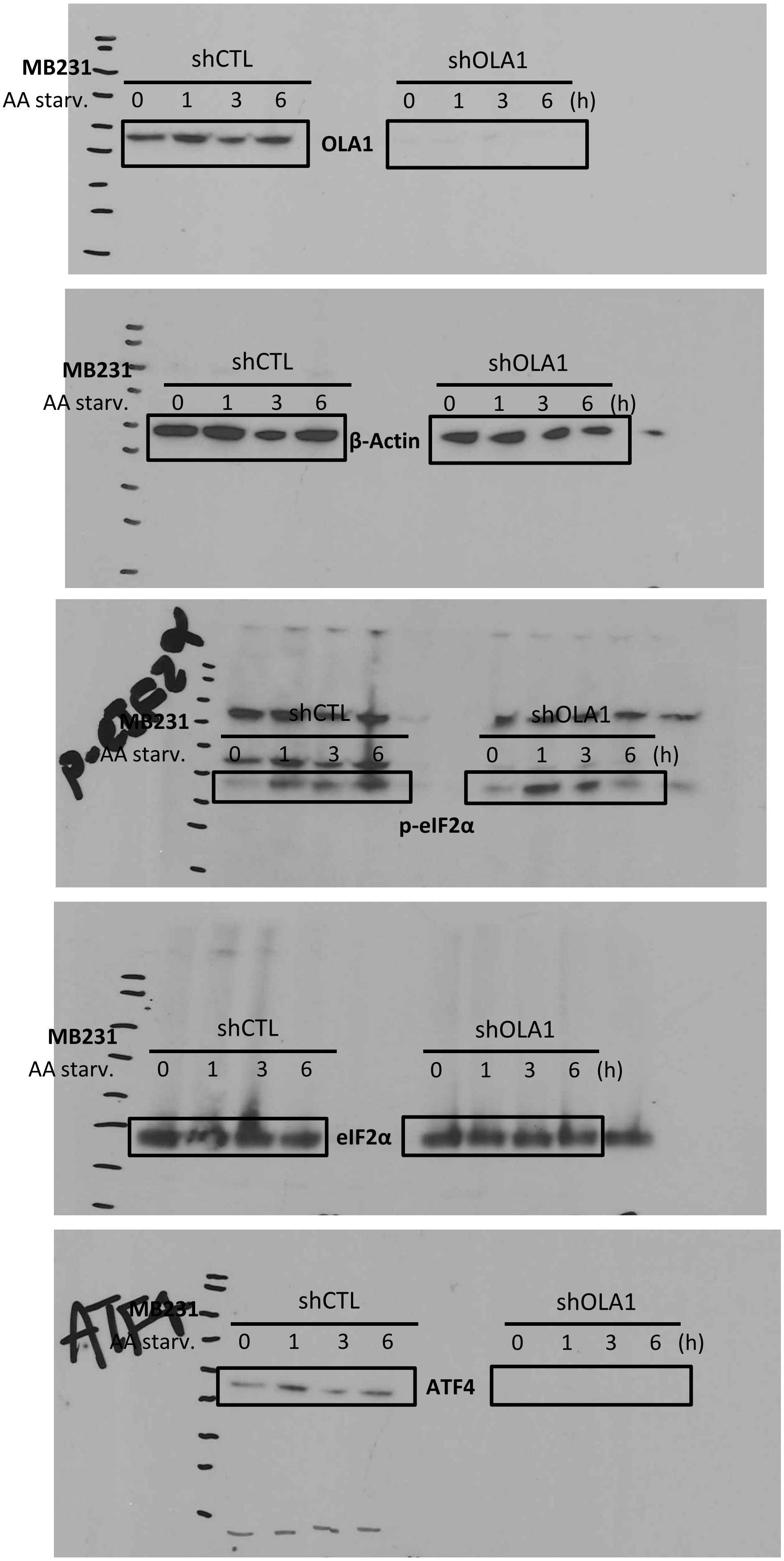

MB231
AA starv.
shCTL
shOLA1
0 1 3 6
0 1 3 6 (h)
OLA1
β-Actin
p-eIF2α
eIF2α
ATF4

Figure 3B

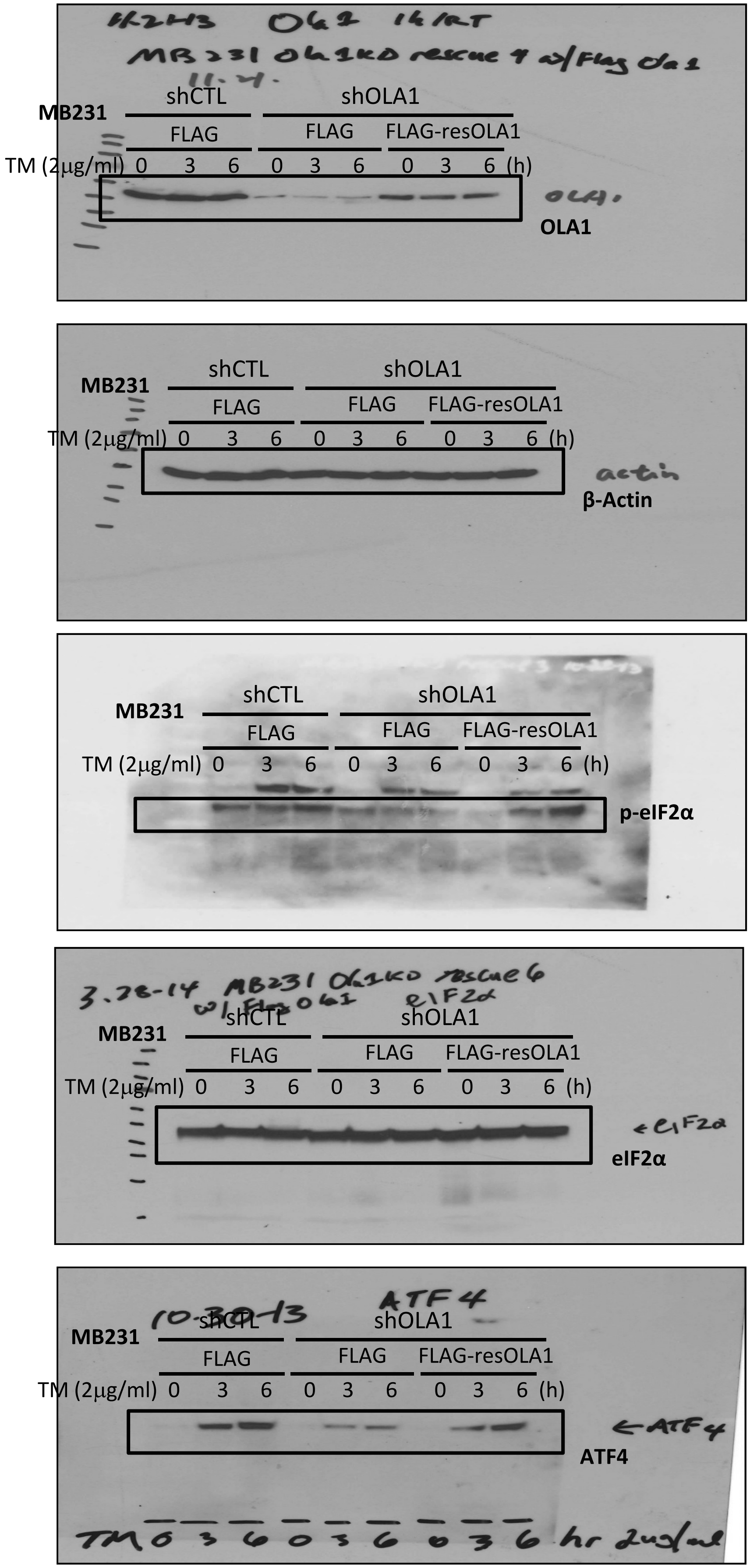
MB231
shCTL
shOLA1
FLAG
FLAG
FLAG-resOLA1
TM (2μg/ml) 0 3 6 0 3 6 0 3 6 (h)
OLA1
β-Actin
p-eIF2α
eIF2α
ATF4

Figure 3C

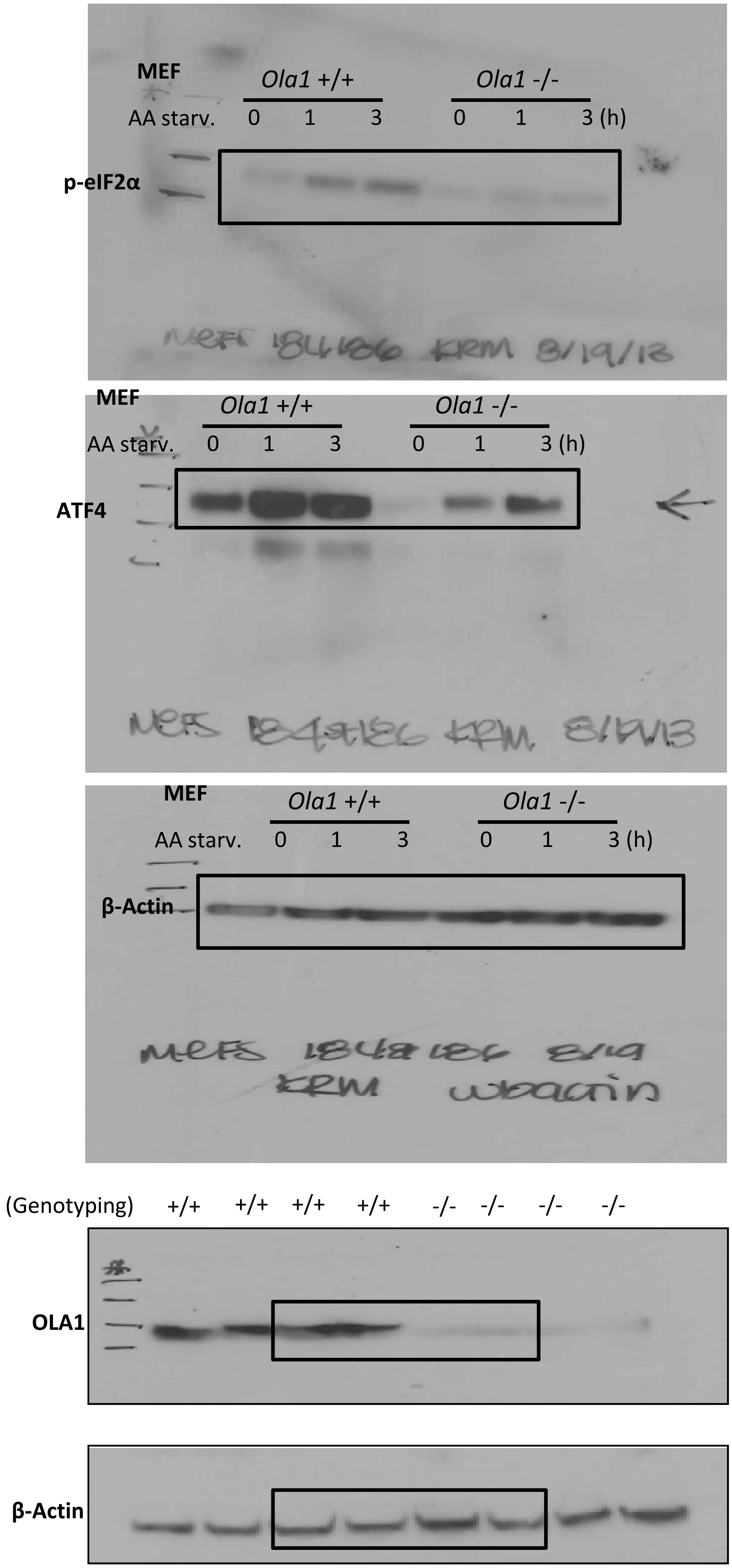

Supplementary Figure 12. Full scan images of immunoblots presented in Figures 3A-C.

Figure 5D

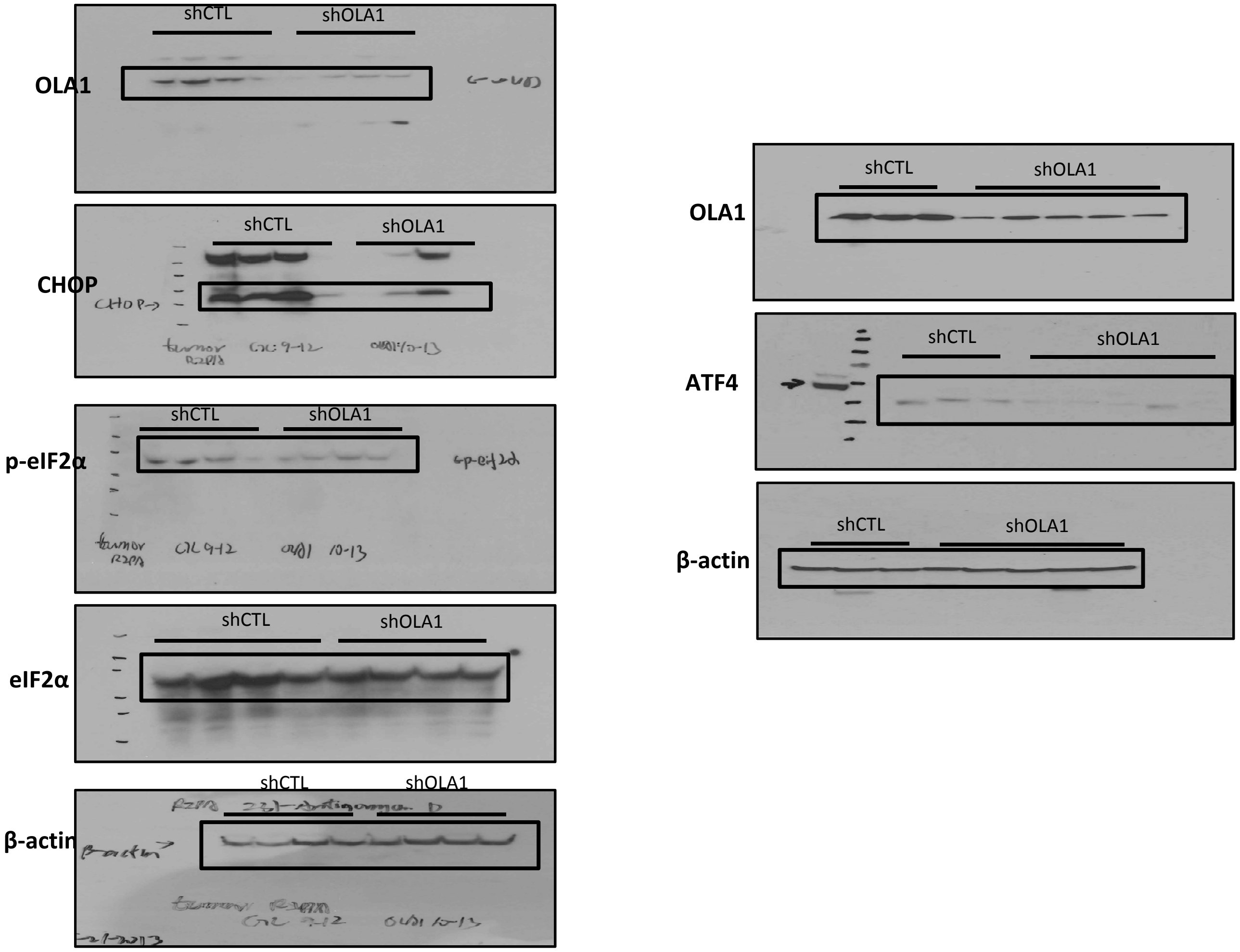

Supplementary Figure 13. Full scan images of immunoblots presented in Figures 5D.

Supplementary Table

Supplementary Table 1. Correlation analysis between OLA1 expression and pathoclinical characteristics in breast cancer.

Clinicopathological parameters	n	OLA1 score 0	1	2	3	χ2	p
Age(yr)	160	18	61	58	23	3.784	0.286
<50	72	6	25	27	14		
>=50	88	12	36	31	9		
Histological type	157*	17	60	58	22	1.97	0.922
Invasive ductal carcinoma	112	11	44	40	17		
Simple carcinoma	24	4	9	9	2		
Others	21	2	7	9	3		
pT	152*	17	57	55	23	10.557	0.103
1	42	3	18	11	10		
2	91	12	36	34	9		
3	19	2	3	10	4		
pN	160	18	61	58	23	4.823	0.567
0	82	11	28	30	13		
1	44	4	19	13	8		
2-3	34	3	14	15	2		
ER	82*	8	35	28	11	6.28	0.099
0	39	3	19	12	5		
1	43	5	16	16	6		
PR	82*	8	35	28	11	3.359	0.34
0	43	5	19	15	4		
1	39	3	16	13	7		

* Total available cases with the particular parameter

Supplementary Methods

Antibodies

Antibodies used in this study including p-Akt (Thr308) (Cat. #2965), Akt (4691), GSK-3α (4337), p-GSK3β (9323), GSK-3β (9315), Snail (3879), β-catenin (9587), Vimentin (3932), MTA1 (5646), BCL2 (2876), BAD (9292), Survivin (2803), C-myc (9402), p-4EBP-1 (2855), 4EBP-1 (9644), p-eIF2α (3398), eIF2α (5324), ATF4(11815), cyclin D3(2936), eIF4E(2067), Gab2 (3239), CHOP (2895), PERK (5683), PCNA (2586), FAK (3285), p70S6 Kinase (9202), p-p70S6 Kinase (Thr389)(9205), RPS6 (2317S), RPL26 (5400S) and Cyclin D1 (2926) were purchased from Cell Signaling Technology. Anti-OLA1 antibody was purchased from Abcam (ab51077) or Sigma-Aldrich (HPA035790). Anti-Ki67 (PA1-38032) antibody was from Thermo Scientific; Anti-CD31 antibody was from Abcam (ab28364); Anti-β-actin (012M4821) antibody was from Sigma-Aldrich. Antibodies against eIF2β (sc9978), GCLM (sc-22754) and FLAG tag (129K4754) was from Santa Cruz Biotechnology. Anti-eIF2γ antibody (N1C1) was from GenTex. Secondary antibodies, including anti-mouse IgG peroxidase linked whole antibody (NXA931) and anti-rabbit IgG peroxidase linked whole antibody (NA934V) were from GE Healthcare; Anti-HA antibody was from Roche Applied Science (#14034800).

Cell treatment and viability assay

Cells were seeded in a microplate at $1x10^4$ cells/well. On the next day, cells were treated with chemical agents as indicated in Results. Cell viability was evaluated by the standard MTT assay or with the MTS-based CellTiter 96 Aqueous One Solution Reagent (Promega). For serum starvation, partial amino acid starvation, total amino acid starvation, and glucose deprivation experiments, the cells were washed with PBS and changed to the serum-free DMEM medium, DMEM without methionine and cysteine (Invitrogen) plus dialyzed fetal bovine serum (dFBS, Invitrogen), the Krebs-Ringer bicarbonate buffer (Sigma-Aldrich) plus dFBS, or DMEM without glucose (Invitrogen), respectively, for the indicated time. For hypoxia treatment, the plates were placed in a hypoxia incubator (Thermo Scientific) at 1% oxygen concentration for 48 h.

In vitro eIF2α phosphorylation and dephosphorylation assay

In vitro eIF2α phosphorylation assay was performed in 25µl reaction buffer (40 mM Tris-HCl at pH7.5, 1 mM Mg Acetate, 80 mM KCl, 0.1%BSA, 100 µM ATP and 1 mM DTT) containing 5 µl of the Rabbit Reticulocyte Lysate (Untreated, Promaga), 400 µM of recombinant human OLA1 protein or its mutant forms, or the control RFP, with or without 30 ng of recombinant PERK with the activity of 18 nmol/min/mg (SignalChem). After incubated at 30°C for 10 min, reactions were stopped on ice and subjected to immunoblot analysis. Phosphorylation of eIF2α was detected by anti-p-eIF2α antibody.

For analyzing eIF2α dephosphorylation *in vitro*, we first prepared phosphorylated eIF2 by incubating the eIF2 holoprotein purified from rabbit RRL (20 µg), a kind gift from Dr. William Merrick of Case Western Reserve University, with 1 µg PKR (SignalChem) in a 200 µl reaction buffer (1 mM ATP, 2 mM Mg acetate, 1 mM DTT, 20 mM Tris-HCl at pH 7.4, and 40 mM KCl) at 37°C for 40 min. The reaction was stopped by adding EDTA to a final concentration of 10 mM. The massive formation of eIF2α-P was confirmed by sampling and immunoblotting analysis. The dephosphorylation assay was performed by incubating an aliquot of the phosphorylation product (that contained ~ 40 nM of total eIF2) with 1.25 units of the recombinant protein phosphatase 1 (PP1, New England Biolabs) in the presence or absence of the recombinant OLA1-WT protein (100 nM) in 100 µl buffer containing 20 mM Tris-HCl (pH 7.4), 50 mM KCl, 2 mM MgCl2, 1 mM DTT, 0.1 mM EDTA and 0.025% Tween 20, at 37°C for 5, 15, and 30 min, and followed by SDS-PAGE gel separation and immunoblotting analysis.

Immunoprecipitation (IP), *in vitro* binding, and immunoblot analysis

Cells were harvested in ice-cold PBS (pH 7.4) and lysed in the lysis buffer containing 25 mM Tris-HCl (pH 7.4), 150 mM NaCl, 1 mM EDTA, 1% NP-40, 5% glycerol supplemented with protease inhibitor cocktail (Thermo Scientific) and phosphatase inhibitors (Sigma-Aldrich). Protein concentrations were determined by the Bradford protein assay (Bio-Rad). For IP of *in vivo* proteins, cell lysates were incubated with the indicated antibodies at 4°C for 3 h to overnight. Protein A/G plus agarose (Santa Cruz Biotechnology) was then incubated with immunocomplexes overnight and washed six times with ice-cold lysis buffer. In the case of IP of FLAG-tagged proteins, anti-FLAG M2 magnetic beads (Sigma-Aldrich) were used according to the manufacturer's instructions. To verify protein–protein interaction *in vitro*, the recombinant HIS-tagged eIF2α (280 nM, Enzo life sciences) and OLA1 (500 nM) proteins were incubated in the assay buffer [25 mM Tris, (pH7.5) 150 mM NaCl, and 0.5 mM EDTA, 1% NP40 and 5% Glycerol] at 4°C for 3 h, followed by the immunoprecipitation with the anti-eIF2α antibody (Cell Signaling) or the normal Rabbit IgG antibody (Santacruz Biotech). Alternatively, purified eIF2 was incubated with recombinant His-tagged OLA1 or the control His-tagged RFP, and the *in vitro* pull-down assay was done with the Dynabeads His-Tag Isolation & Pulldown beads (Life Technologies). For all immunoblotting analyses, proteins from cell lysate (~ 50 µg) or the IP products were dissolved in sample loading buffer (Thermo Scientific) and heated at 100°C for 10 min before separated with a 4-20% gradient polyacrylamide gel (Invitrogen) and transferred to a nitrocellulose membrane (PVDF, Bio-Rad). The membrane was block with 5% nonfat milk and incubated subsequently with primary antibody and secondary antibody. The immunoreactive bands were visualized using a chemiluminescence detection system (GE Healthcare). To extract proteins from frozen tumor tissues, the tissues were first broken and grinded with a mortar and pestle in liquid nitrogen, and then lysed in RIPA buffer (Sigma-Aldrich) with brief solicitations on ice.

Immunohistochemical (IHC) Analysis

For IHC staining of xenograft tumors harvested from the mouse models, 5 µm-thick sections from paraffin-embedded tissue mounted on coated glass slides were deparaffinized, hydrated, and heated in 10 mM sodium citrate buffer (pH 6.0) for 15 min in a steamer for antigen retrieval. After brief treatment with the Background Buster blocking agent (Innovex Biosciences, Richmond, CA), the slides were incubated with the diluted primary antibody for 45 min. After washing with the Innovex washing buffer, the slides were incubated sequentially with STAT-Q secondary linking antibody (Innovex Biosciences), HRP-labeled streptavidin, and the Innovex substrate (DAB) for color development, following the manufacturer's instructions.

One hundred sixty human breast cancer specimens in paraffin blocks were made into tissue arrays using a ZM-1 tissue array machine [3]. Sections (4-µm thick) were cut and immunostained for OLA1-expression. The IHC was evaluated independently by two experienced pathologists. Protein expression levels were determined semi-quantitatively by combining the proportion and intensity of the positively stained tumor cells. The percentage of positive cells (0: <5%, 1: 5%-25%, 2: 26%-50%, 3: 51%-75%, and 4: >75%) and the staining intensity (0: negative, 1: weak, 2: moderate, and 3: strong), were summed to give overall scores of the expression levels of OLA1: 0 (0-1, negative expression), 1 (2-3, low expression), 2 (4-5, medium expression), and 3 (6-7, high expression).

Supplemental Reference

1. Cerami, E. *et al.* The cBio cancer genomics portal: an open platform for exploring multidimensional cancer genomics data. *Cancer discovery* **2**, 401-404 (2012).
2. Gao, J.J. *et al.* Integrative Analysis of Complex Cancer Genomics and Clinical Profiles Using the cBioPortal. *Sci Signal* **6** (2013).
3. Meng, P. Q. *et al.* Application of new tissue microarrayer-ZM-1 without recipient paraffin block. *Journal of Zhejiang University. Science. B* **6**, 853-858, doi:10.1631/jzus.2005.B0853 (2005).
